# Integrated Analysis of Competitive Endogenous RNA Networks in Acute Ischemic Stroke

**DOI:** 10.3389/fgene.2022.833545

**Published:** 2022-03-25

**Authors:** Zongkai Wu, Wanyi Wei, Hongzhen Fan, Yongsheng Gu, Litao Li, Hebo Wang

**Affiliations:** ^1^ Department of Neurology, Hebei Medical University, Shijiazhuang, China; ^2^ Department of Neurology, Hebei General Hospital, Shijiazhuang, China

**Keywords:** acute ischemic stroke, differentially expressed genes, differentially expressed miRNAs, functional enrichment analyses, competitive endogenous RNA, protein-protein interaction, qPCR analysis

## Abstract

**Background:** Acute ischemic stroke (AIS) is a severe neurological disease with complex pathophysiology, resulting in the disability and death. The goal of this study is to explore the underlying molecular mechanisms of AIS and search for new potential biomarkers and therapeutic targets.

**Methods:** Integrative analysis of mRNA and miRNA profiles downloaded from Gene Expression Omnibus (GEO) was performed. We explored differentially expressed genes (DEGs) and differentially expressed miRNAs (DEMirs) after AIS. Target mRNAs of DEMirs and target miRNAs of DEGs were predicted with target prediction tools, and the intersections between DEGs and target genes were determined. Subsequently, Gene Ontology (GO) and Kyoto encyclopedia of genes and genomes (KEGG) pathway enrichment analyses, Gene set enrichment analysis (GSEA), Gene set variation analysis (GSVA), competitive endogenous RNA (ceRNA) (lncRNA-miRNA-mRNA) network, protein–protein interaction (PPI) network, and gene transcription factors (TFs) network analyses were performed to identify hub genes and associated pathways. Furthermore, we obtained AIS samples with evaluation of immune cell infiltration and used CIBERSORT to determine the relationship between the expression of hub genes and infiltrating immune cells. Finally, we used the Genomics of Drug Sensitivity in Cancer (GDSC) database to predict the effect of the identified targets on drug sensitivity.

**Result:** We identified 293 DEGs and 26 DEMirs associated with AIS. DEGs were found to be mainly enriched in inflammation and immune-related signaling pathways through enrichment analysis. The ceRNA network included nine lncRNAs, 13 miRNAs, and 21 mRNAs. We used the criterion AUC >0.8, to screen a 3-gene signature (FBL, RPS3, and RPS15) and the aberrantly expressed miRNAs (hsa-miR-125a-5p, hsa-miR-125b-5p, hsa-miR-148b-3p, and hsa-miR-143-3p) in AIS, which were verified by a method of quantitative PCR (qPCR) in HT22 cells. T cells CD8, B cells naïve, and activated NK cells had statistical increased in number compared with the acute cerebral infarction group. By predicting the IC50 of the patient to the drug, AZD0530, Z.LLNle.CHO and NSC-87877 with significant differences between the groups were screened out. AIS demonstrated heterogeneity in immune infiltrates that correlated with the occurrence and development of diseases.

**Conclusion:** These findings may contribute to a better understanding of the molecular mechanisms of AIS and provide the basis for the development of novel treatment targets in AIS.

## Introduction

Stroke is one of the leading causes of permanent disability worldwide and among the leading causes of mortality. Approximately nine million people worldwide suffer from stroke for the first time each year, and approximately 6.5 million people have long-term disabilities (Dennis et al., 2019). Acute ischemic stroke (AIS) is triggered by obstruction of blood flow to the local brain due to a clot or embolus blocking a cerebral artery (Adams et al., 1993; Fatahzadeh and Glick, 2006; Benjamin et al., 2018). In the ischemic stroke, a complex pathophysiological cascade is strongly correlated, both spatially and temporally, with the reduction of cerebral blood flow (Dirnagl et al., 1999). The pathogenesis of AIS includes ischemic brain injury caused by a chain of events (ischemic cascade) that are triggered by secondary injuries occurring hours or days after the initial event (Iadecola et al., 2020). However, the molecular mechanisms of ischemic stroke remain unclear. Therefore, to improve diagnosis and treatment, the molecular mechanism of ischemic stroke needs to be investigated. Early diagnosis and successful treatment beneficial in minimizing the damage to the brain, thus reducing mortality and improving prognosis. The current diagnosis of stroke has been severely hampered by the lack of rapid, valid, and analytically sensitive diagnostic biomarkers (Saenger and Christenson, 2010). Neuroimaging remains the most reliable tool for the diagnosis of ischemic stroke. Thrombolytic treatment through the tissue plasminogen activator (tPA) agent and surgical removal of clots represent the current therapeutic approaches for the treatment of AIS. While these therapies can restore cerebral blood flow and are efficient treatments for AIS, therapy designed to simultaneously target multiple mechanisms of cell injury is needed. Thus, to achieve improved clinical efficacy, there is an urgent need for novel biomarkers with high sensitivity and specificity for early diagnosis and treatment of ischemic stroke.

LncRNAs are non-coding RNAs with more than 200 nucleotides in length and lacking of the protein coding potential (Karagkouni et al., 2020). LncRNA molecule serves as a “sponge” and is capable to compete for miRNA binding (Karagkouni et al., 2020). Current evidence has shown that the expression and function of miRNA can exert either pro-inflammatory or anti-inflammatory effect after ischemic stroke, and that miRNAs are negatively regulated by lncRNAs. LncRNA SNHG14, which acts as a competitive sponge for miR-136–5p, miR-145–5p, and miR-199b, regulates the activation of microglia and exhibits pro-inflammatory ability (Qi et al., 2017; Zhong et al., 2019; Zhang G. et al., 2021). In contrast to the pro-inflammatory properties of the M1 microglia, the M2 microglia is responsible for the removal of debris and facilitating tissue repair through anti-inflammatory factors primarily at the recovery stage (Hu et al., 2015). Knockdown of lncRNA H19 can negatively regulate the expression of miR-29b and miR-138–5p, and therefore, can promote functional recovery after cerebral ischemia and the polarization of microglia (Li et al., 2020; Xu J. et al., 2021). A recent study shows that lncRNA MALAT1, sponging miR-30a, promotes neuronal cell death and suppresses autophagy in ischemic stroke (Guo et al., 2017). Studies have shown that miR-145 plays an essential role in inflammation after ischemic stroke. A previous study also indicated that the production of inflammatory cytokines is regulated by lncRNA TUG1 at an early stage after ischemic injury by targeting miR-145a-5p (Wang et al., 2019). Enhancing our understanding of the interactions between RNAs through competitive endogenous RNA (ceRNA) network can elucidate new AIS-related molecular mechanisms and identifying novel biomarkers for AIS. Thus, elucidation of the mechanistic details of AIS occurrence and progression, and exploration of potential biomarkers and therapeutic targets are critical to developing new treatments and diagnostics.

Neuroinflammation is driving cause of the pathophysiological processes leading to ischemic stroke (Doll et al., 2014; Zhao et al., 2016). Several pathophysiological processes could negatively affect homeostasis of physiological functions, including excitotoxicity, excessive formation of reactive oxygen species (ROS), loss of glucose, and oxygen mitochondrial dysfunction, neuronal apoptosis, and blood-brain barrier permeability (Forrester et al., 2018; Chen W. et al., 2020; Xu Q. et al., 2021). Local and peripheral immune system plays important roles in the pathophysiology of stroke, and includes both the innate and the adaptive immune responses (Benakis et al., 2016; Venkat et al., 2018). Neurotoxic factors including reactive oxygen and nitrogen species as well as exopeptidases can be released immediately after peripheral immune system contribute to secondary neurodegeneration (Benakis et al., 2016). Immune cells, including microglia, monocyte/macrophages, neutrophils, and lymphocytes infiltrate into the brain after stroke and induce inflammatory or anti-inflammatory responses via distinct pathways (Zhang S. R. et al., 2021).

Two studies (Zhang et al., 2020; Sun et al., 2021) aimed to explore possible molecular mechanisms of ischemic stroke by constructing ceRNA networks. Our study used different datasets from that of Sun et al. The study of Sun et al. included the miRNA expression profile of GSE55937, the mRNA and lncRNA expression profile of GSE122709, and the mRNA expression profile of GSE146882. However, our study included the mRNA expression profiles of GSE16561, and the miRNA dataset GSE110993. These two studies (Zhang et al., 2020; Sun et al., 2021) only performed ceRNA network analysis, while our study used the hypergeometric distribution model to evaluate the ceRNA interaction network and performed ROC analysis for key genes. Relative to these two studies, we added GSEA analysis, GSVA analysis, TFs network analysis, immune infiltration analysis and drug network analysis. The ceRNA network we found includes 9 lncRNAs such as AL360004.1, LINC00173, LINC01089, LINC00115, 13 miRNAs such as hsa-miR-125a-5p, hsa-miR-125b-5p, hsa-miR-148b-3p and hsa-miR-143-3p and 21 mRNAs such as FBL, RPS3, RPS15. The difference was that the ceRNA network constructed by Sun et al.'s study contains 7 mRNAs, 14 lncRNAs, such as SND1-IT1, NAPA-AS1, LINC01001, LUCAT1, ASAP1-IT2, 8 miRNAs, such as miR-93 -3p, miR-24-3p. Zhang et al. found that MCM3AP-AS1, LINC01089, ITPK1-AS1 and HCG27 may be new biomarkers and potential targets for AIS therapy.

Finally, we determined the DEGs and DEMirs in AIS through a comprehensive analysis. Gene expression profiles in cerebral infarction samples were obtained through the GEO database. Subsequently, GO, KEGG, GSEA, and GSVA were used to study the molecular mechanisms for DEGs in AIS. Then, a ceRNA network and TFs network were established. Receiver operating characteristic (ROC) curve analysis was implemented to explore the diagnostic validity of the identified DEMirs and DEGs. We identified three potential mRNAs and four potential miRNAs as important predictors of AIS. Moreover, we further conducted immune infiltration analysis and drug sensitivities of the cell lines expressed, as the half maximal inhibitory concentration (IC50). Our research will clarify the molecular mechanisms of AIS and provide the basis for new applications in both diagnosis and treatment.

## Materials and Methods

### Data Download and Data Pre-Processing

**FIGURE 1:**
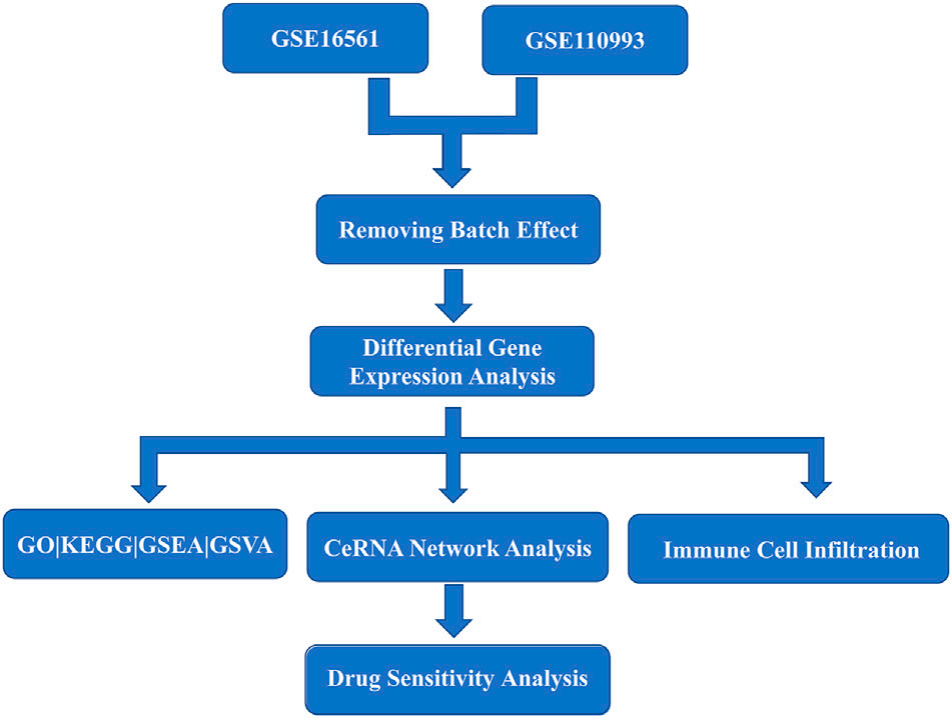
Flow chart of overall analysis.

Acute cerebral infarction expression profile datasets GSE16561 (Barr et al., 2010) and GSE110993 (Tiedt et al., 2017) with reliable sample sources were downloaded from the GEO (https://www.ncbi.nlm.nih.gov/geo/) database by using the GEO query package (Davis and Meltzer, 2007)of R software (version 3.6.6). The samples in the datasets are all from *Homo sapiens*. The data in the two datasets were generated using different platforms: GPL6883 Illumina HumanRef-8 v3.0 expression beadchip and GPL15456 Illumina HiScanSQ respectively. GSE16561 dataset includes whole blood samples from 39 patients with acute cerebral infarction and 24 healthy controls. The GSE110993 dataset includes whole blood samples from 20 patients with acute cerebral infarction and 20 healthy patients for inclusion. The raw data were converted into an expression matrix and corrected for background and normalized by the limma package (Ritchie et al., 2015). Afterwards, the batch effect was removed in sva package (Leek et al., 2012). A flow diagram for the present analysis is shown in Figure 1.

### Data Analysis: Differential Expression and Pathway Analyses

First, Principal Component Analysis (PCA) was conducted using FactoMineR package (Lê et al., 2008). Then, DEGs were identified using the limma package (Ritchie et al., 2015), and volcano plots of DEGs were generated using the ggplot2 package (Walter et al., 2015). Finally, adjusted *p* < 0.05 and |log2FC| > 0.5 were used as the cutoff criteria to identify DEGs. We performed GO and KEGG enrichment analysis using the clusterProfiler R package (Yu et al., 2012), and differences were considered statistically different at adjusted *p* value <0.05.

### GSEA Analysis, GSVA Analysis

GSEA was performed on the gene expression matrix using clusterProfiler R package (Yu et al., 2012), and enrichment was considered significant for false discovery rate (FDR) < 0.25 and *p* < 0.05. The gene sets were analyzed using the gene matrix transposed (gmt) file downloaded from MSigDB. Each gene set was constructed into a GSVA score matrix. Then, according to the GSVA scores, the gene sets were separated into low- and high-score groups. The limma R package was applied to define the significant differences between the low- and high-score groups. Finally, heat maps were drawn using the R package pheatmap.

### ceRNA Interaction Network Analysis

Normalized data were analyzed using the GDCRNATools package (Li et al., 2018), and then screened for differential mRNAs, miRNAs, and lncRNAs. The miRcode database (Jeggari et al., 2012) was respectively utilized to pair lncRNA-miRNA and mRNA-miRNA. The hypergeometric distribution model was constructed to evaluate the ceRNA interaction network, and finally visualization was performed with Cytoscape (Shannon et al., 2003).

### ROC Analysis of Key Molecules

A pROC package (Robin et al., 2011) was used to perform the leave-one-out jackknife approach and draw the ROC curve of key molecules, with the sensitivity as the ordinate and 1-specificity as the abscissa. The area under the curve (AUC) served as the main evaluation performance. The higher the AUC value, the better is the predictive power.

### Immune Cell Infiltration Level, Correlation Analysis of Immune Cells

CIBERSORT (Newman et al., 2015) is a tool used for deconvolution of the transcriptome expression matrix based on the principle of linear support vector regression, which can estimate the composition and abundance of infiltrating immune cells of the acute cerebral infarction sample in the mixed cells. After uploading the gene expression matrix data to CIBERSORT (Newman et al., 2015) and filtering the outputs (*p* < 0.05), we obtained the immune cell infiltration matrix and the immune infiltration distribution results of acute cerebral infarction. The resulting correlation of 21 types of infiltrated immune cells was visualized in a heat map format generated by the corrplot package (Friendly, 2002) of R.

### The Upstream Transcription Factor Network That Regulate miRNAs and Drug Network

Prediction of transcription factors regulating differentially expressed genes was analyzed by FunRich software (Pathan et al., 2015). By selecting the intersection molecules of the predicted transcription factor, mRNA, and miRNA target genes, we reconstructed the predicted transcription factor-miRNA regulatory networks. In addition, Genomics of Drug Sensitivity in Cancer (GDSC database) (Yang et al., 2013), that covers the sensitivity and response of cells to drugs, was employed. For further network pharmacology analysis, we predicted the IC50 value and compared the *p*-value of the rank sum test between acute cerebral infarction and normal samples to determine drug sensitivity.

### Cell Culture and Treatment

The immortalized mouse hippocampal neuronal cell line, HT22 (Zhejiang Ruyao Biotechnology Co. Ltd., Zhejiang, China), was cultured in Dulbecco’s modified Eagle’s medium (DMEM, Corning, NY, United States) containing 1% penicillin/streptomycin and 10% fetal bovine serum (FBS, BI, Israel) at 37°C in a humidified incubator containing 5% CO2. For cell lysis of adherent cells, cells were grown to 60–80% confluence and were rinsed with PBS before trypsinization. Thereafter, HT22 cells were randomly divided into the normal group and the model group. Cells were seeded in a 96-well plates at a density of 5 × 104 cells/well and cultured for 24 h. After 24 h, the culture medium was discarded, and the cells of model group were washed twice with PBS. Serum and glucose free media were used, and cells were placed in an anaerobic culture box inside the 37°C incubator for 4 h. After hypoxia, glucose free media was replaced with complete DMEM and the cells were reoxygenated in a normoxic incubator at 37°C. These cells of control group were cultured at 37°C in a 5% CO2 incubator under normal atmospheric oxygen conditions. Cell viability from the model and control groups was determined using the Cell Counting Kit-8 assay (CCK-8, Biosharp, China).

### Quantitative Polymerase Chain Reaction (qPCR Analysis)

Total RNA was extracted using the TRIzol (Life Technologies, Carlsbad, CA, United States), following the manufacturer’s instructions. Briefly, HT22 cells were lysed in TRIzol, then 60 μL chloroform was added, samples were shaken for 1 min, incubated at room temperature for 5 min and centrifuged for 15 min at 12000 g at 4°C. The aqueous phase was transferred into a new tube and RNA was precipitated in the presence of isopropanol. After centrifugation, the supernatant was discarded and the pellet was washed with 200 μL 75% ethanol, made with Diethyl pyrocarbonate (DEPC)-treated water, by centrifugation at 7,500 g, for 10 min at 4°C. RNA was eluted in 20 μL DEPC-treated water, quantified by SmartSpec Plus (Bio-Rad, Hercules, CA) and stored at **−**80°C. Reverse transcription reactions (37°C for 15 min, 85°C for 5 s, 4°C) were carried out using the Evo M-MLV kit (Accurate Biotechnology Co., Ltd, China). The cDNA was synthesized (37°C for 60 min, 85°C for 5 min, 4°C) using the miRNA first-strand cDNA synthesis kit (Accurate Biotechnology Co., Ltd, China). Then, qPCR was performed using the SYBR Green Pro Taq HS qPCR premix and PCR-amplified in a two-step process. The PCR amplification conditions were the following: 40 cycles of 30 s at 95°C, 5 s at 95°C and 30 s at 60°C. The relative expression levels were calculated using the 2^−△△Ct^ method with GAPDH as an internal reference gene. The melting curve was analyzed to assure specificity of the primers after each reaction. See Supplementary Table S1 for primer sequences.

## Statistical Analysis

All analyses were performed using R software for statistical calculations (version 4.0.2). Independent sample *t*-test was used to estimate normally distributed variables whereas Mann-Whitney *U* test was used to compare non-normally distributed variables. The statistical significance of categorical variables was compared using the chi-square test or Fisher exact test. Pearson correlation coefficients were calculated to define the correlation between different genes. ROC curves were generated with the R package pROC, and the corresponding area under curve (AUC) values calculated. All statistical tests were performed two-sided, and statistical significance was set at *p* < 0.05. The results were shown as the mean ± standard deviation (SD).

## Results

### Data Pre-Processing

**FIGURE 2:**
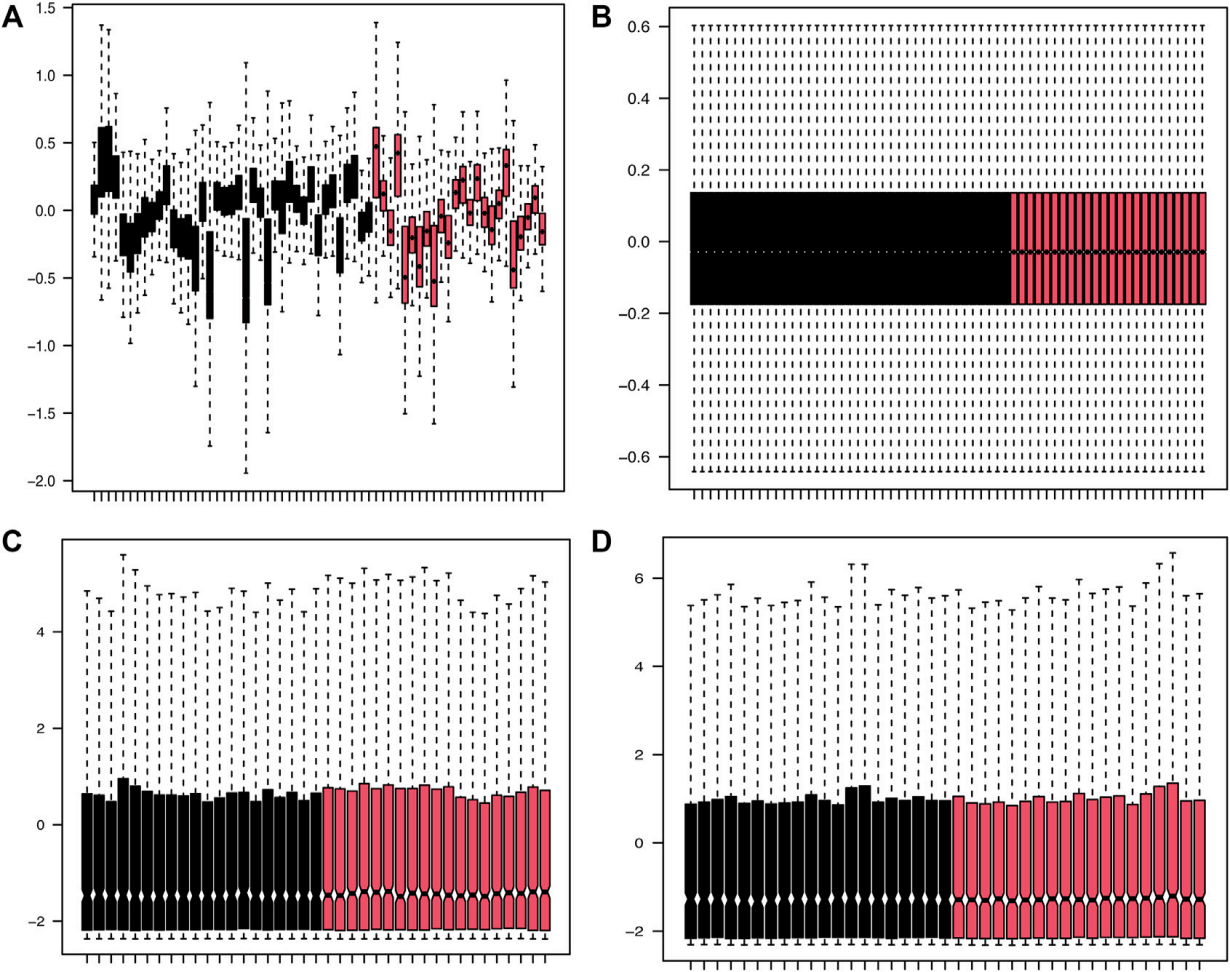
Density plots of the dataset samples before and after correction. **(A,B)** The boxplot of GSE16561 dataset samples before and after correction after removing the inter-batch differences. **(C,D)** The boxplot of GSE110993 dataset samples before and after correction after removing the inter-batch differences.

PData function was available for getting grouping information of the expression profile datasets GSE16561 (Barr et al., 2010) and GSE110993 (Tiedt et al., 2017). Each expression matrix from two raw datasets (GSE16561 and GSE110993) was obtained and then pre-processed identically for background correction and normalization using the limma package (Ritchie et al., 2015). Finally, batch effects were removed using the sva package (Leek et al., 2012). The corresponding boxplot is shown in Figure 2.

### Genes Associated With Cerebral Infarction

**FIGURE 3:**
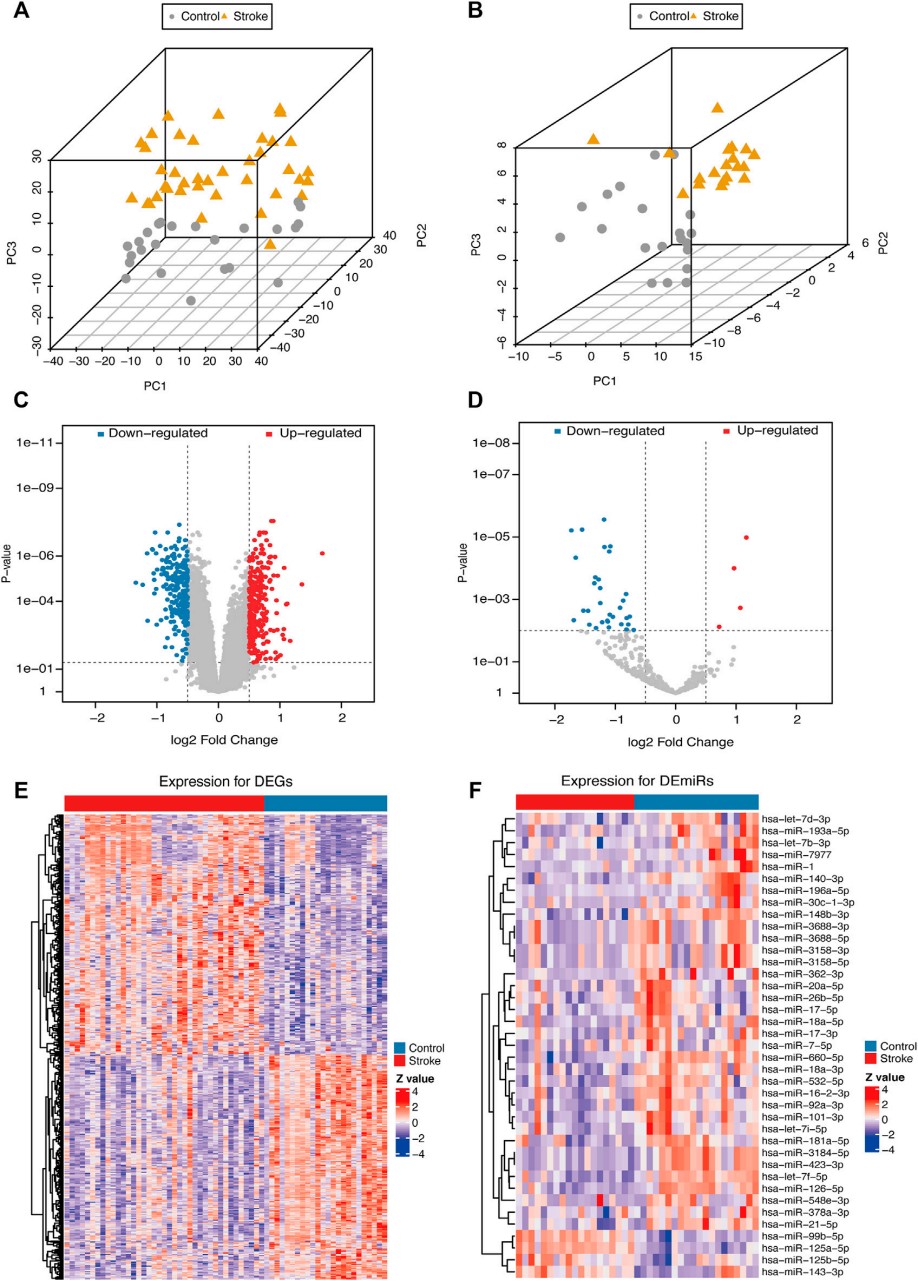
PCA plots and differential expression of the samples of the data sets after correction. PCA plots of the GSE16561 **(A)** and GSE110993 **(B)** datasets after removing the inter-batch differences. Volcano plots of the GSE16561 **(C)** and GSE110993 **(D)** dataset; red plot represented upregulation, and blue plot represented downregulation. Heat maps of the GSE16561 **(E)** and GSE110993 **(F)** datasets. The color scale represented the abundance of gene expression. The darker the color shade, the higher expression level.

After normalization, the gene expression matrices of the two datasets were presented as PCA plots (Figures 3A,B). The results showed that the two groups of samples clustered more obviously after normalization, indicating that the source of the samples was reliable. We used R software to preprocess the data and performed differential expression analysis on the gene expression matrix of GSE16561 data set to obtain 3698 DEGs, while the differential analysis in the GSE110993 data set yielded 62 DEMirs. The results are shown in the volcano plots (Figures 3C,D) and the heat maps (Figures 3E,F).

### Intersected Differentially Expressed Genes and Target Genes

**FIGURE 4:**
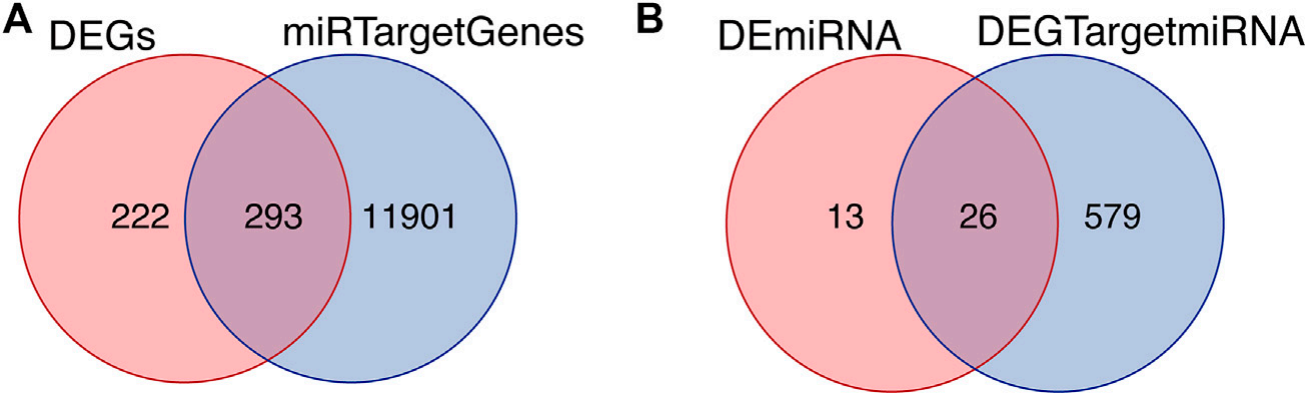
Intersected differentially expressed genes and target genes. **(A)** The intersection of DEGs and miRNA target genes. **(B)** The intersection of differentially expressed miRNAs and target miRNAs of DEGs.

The starbase database, a non-coding RNA database, was used to find target genes based on miRNA, verify the interaction between miRNA and mRNA, and generate a target gene RNA network. Based on the differential expression results and the prediction results of starbase, the intersection of the DEGs and the miRNA target genes was drawn (Figure 4A). The intersection of the differentially expressed miRNAs and target miRNAs of DEGs included 26 genes, as shown in Figure 4B.

### GO/KEGG Enrichment Analysis, Pathway Diagram (Based on DEGs)

**FIGURE 5:**
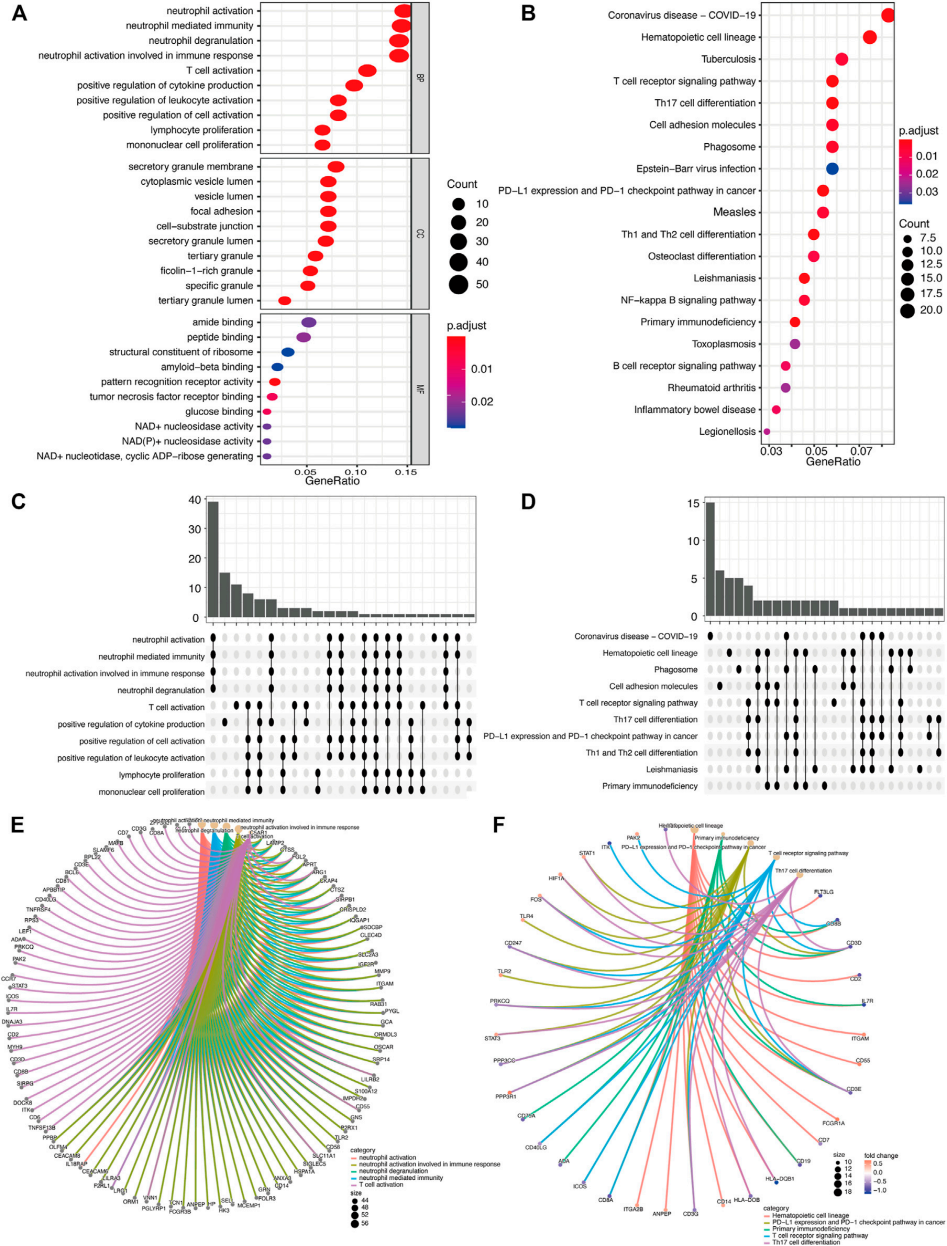
GO/KEGG function enrichment analysis. **(A)** In GO biological function enrichment analysis, the X horizontal axis represents the proportion of DEGs enriched in GO term, and the color of the dot represents the adjusted *p* value: the redder the color, the smaller the adjusted *p* value; the bluer the color, the greater the adjusted *p* value. The size of the dot represents the amount of enriched mRNA. **(B)** In KEGG enrichment analysis, the X horizontal axis represents the proportion of DEGs, and the color of the dot represents the corrected *p* value. **(C)** GO function enrichment analysis upset chart. The horizontal axis represents the categories of term names enriched by DEGs, and the vertical axis represents the number of DEGs in this term. **(D)** KEGG function enrichment analysis Upset plot. **(E)** GO function enrichment analysis circos plot. **(E)** The outer circle is the information of the corresponding entry gene in the enrichment analysis, and the line is the corresponding enrichment term entry. **(F)** KEGG function enrichment analysis circos plot.

**FIGURE 6:**
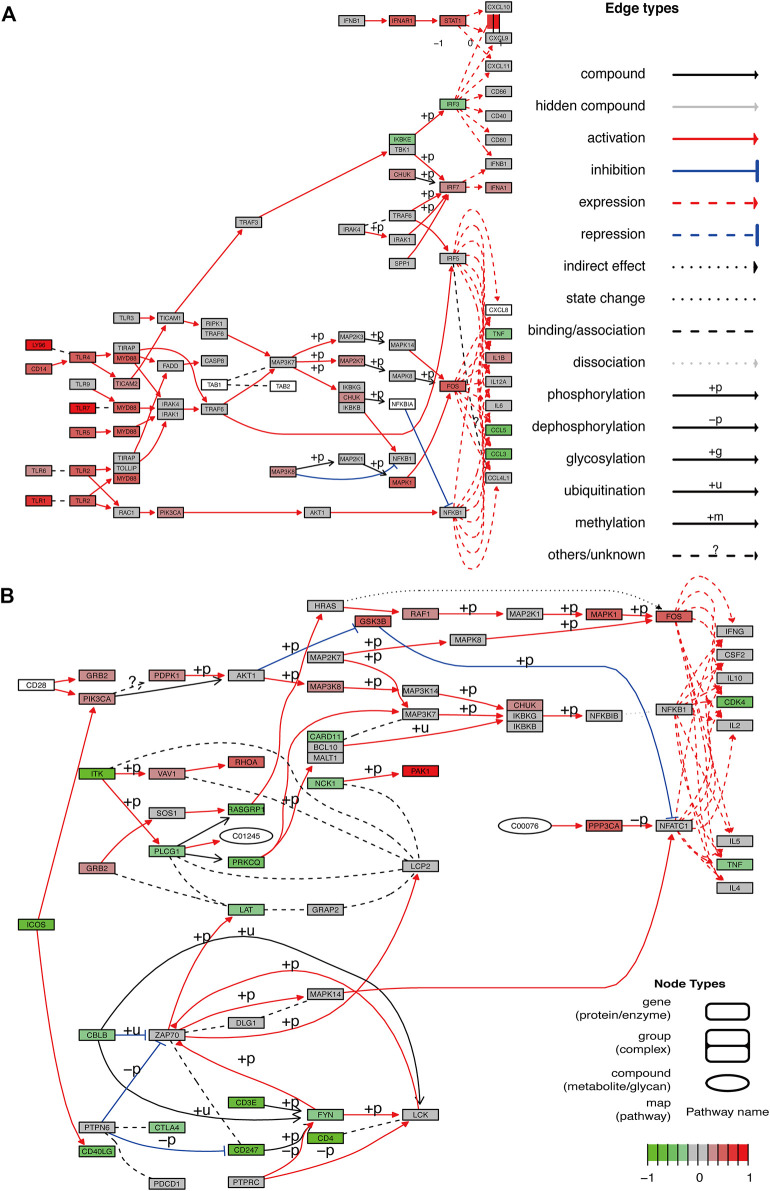
Pathway diagram. **(A,B)** Two pathway diagrams composed of two major networks are constructed using DEGs.

Based on the intersection of the DEGs and the miRNA target genes, GO analysis was performed. The main biological processes involving the DEGs included neutrophil activation, neutrophil degranulation, neutrophil activation involved in immune response. In terms of cellular component, the DEGs were mostly enriched in secretory granule membrane and cytoplasmic vesicle lumen. In terms of molecular functions, the DEGs were linked with amide binding, peptide binding, structural constituent of ribosome, amyloid-beta binding (Figure 5A). As shown in Figure 5B, KEGG analysis results included Coronavirus disease (Covid-19), Hematopoietic cell lineage, Tuberculosis, etc. Figure 5C is an Upset plot of the gene ontology (GO) analysis. The main enrichment can reflect the intersection between different terms. Similarly, Figure 5D is an Upset plot of KEGG data. Figure 5E is a circos diagram of GO analysis based on differentially expressed mRNA. Figure 5F is a circos diagram of KEGG pathway enrichment analysis. The Supplementary Table S2 shows the detailed enrichment analysis results. The two key pathway diagrams were constructed according to the above-mentioned differentially expressed mRNA, as shown in Figure 6.

### GSEA and GSVA

**FIGURE 7:**
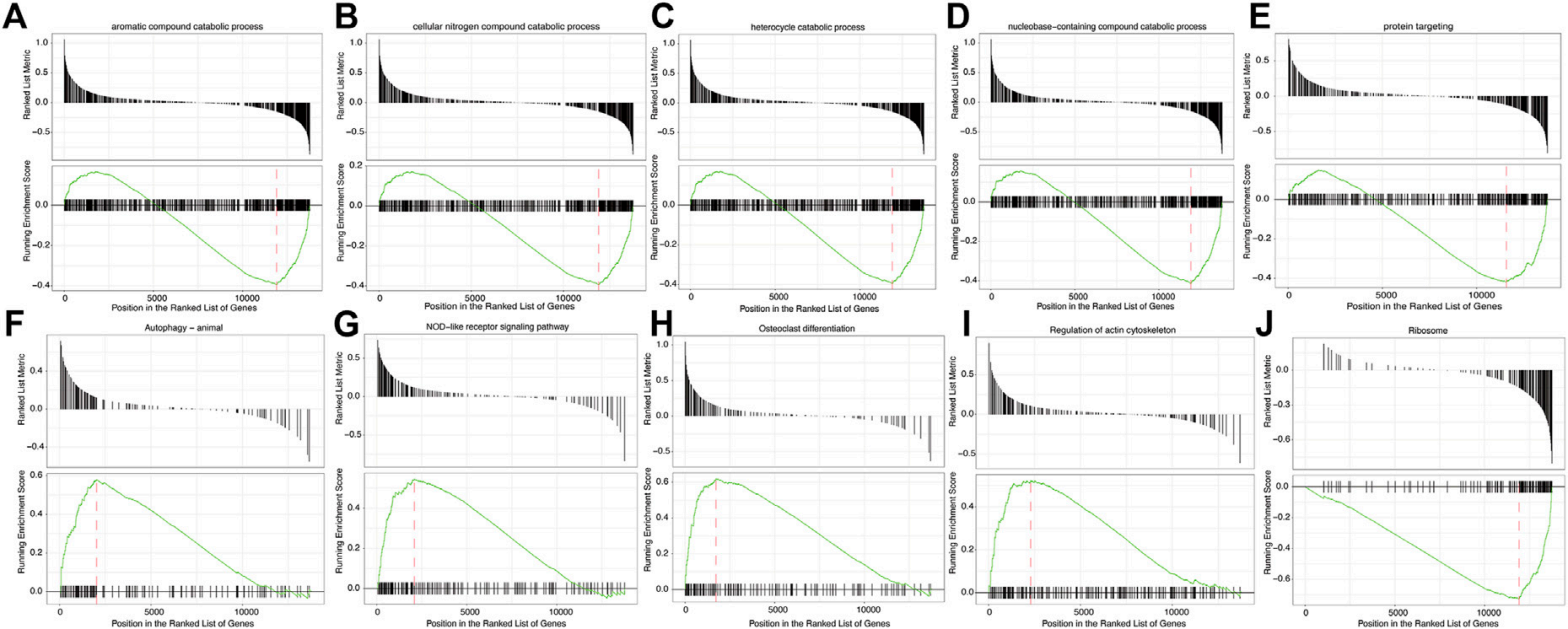
Gene Set Enrichment Analysis (GSEA). **(A**–**J)** GSEA enrichment analysis result sub-graph. The upper part of the graph represents the distribution of rank values of all genes after sorting, and the Signal2Noise algorithm is used by default. The lower part of the graph represents the line chart of the gene Enrichment Score, the horizontal axis is each gene in the gene set, and the vertical axis is the corresponding result.

**FIGURE 8:**
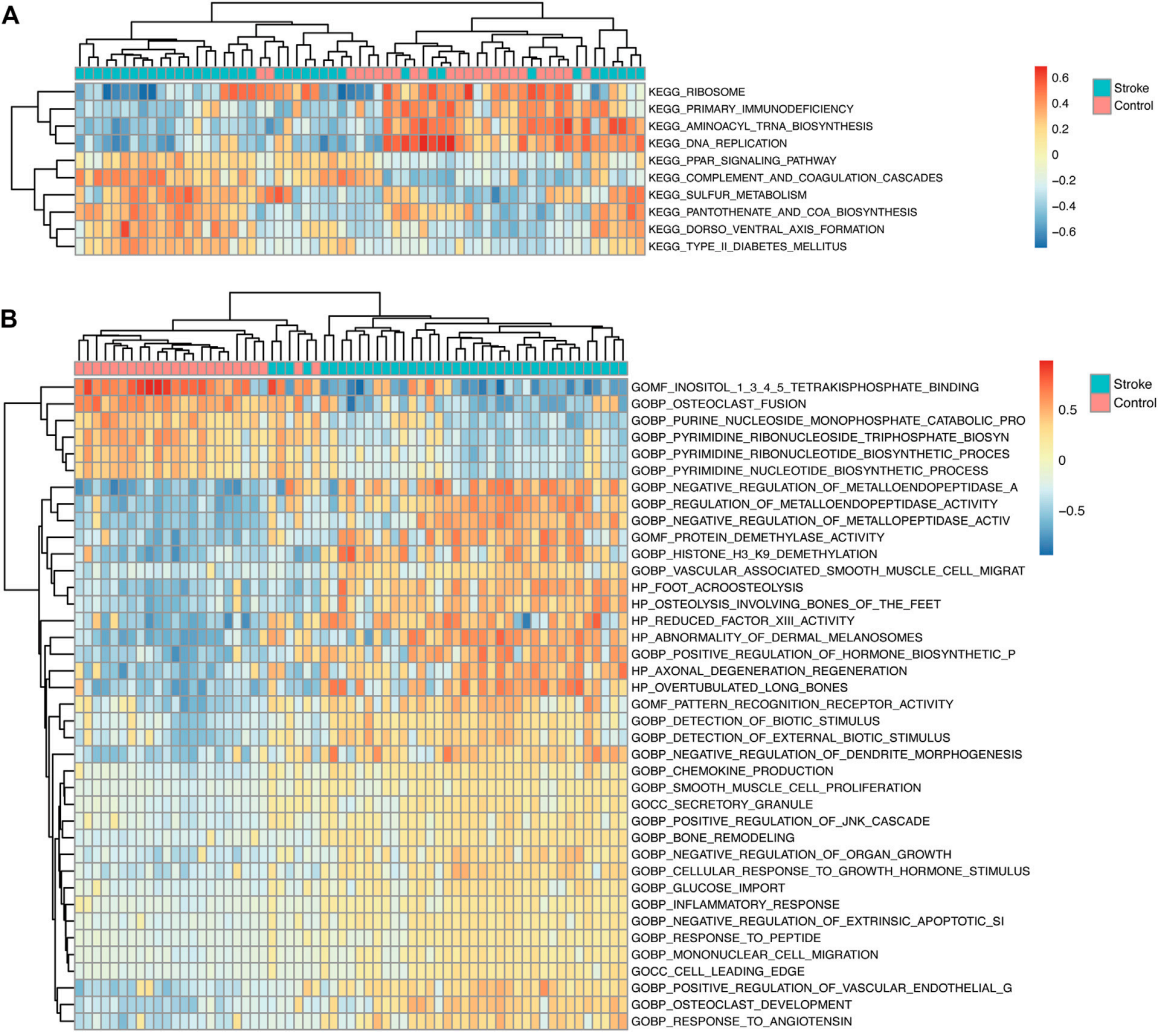
GSVA analysis. **(A)** In the GSVA enrichment analysis of KEGG term entries, the color scale represents the abundance of gene expression, red represents up-regulation, and blue represents down-regulation. The darker the color shade, the higher is the expression level. **(B)** GSVA analysis of GO term entries.

GSEA showed that the pathways were mainly enriched aromatic compound catabolic process, cellular nitrogen compound catabolic process, heterocycle catabolic process, nucleobase-containing compound catabolic process, protein targeting, autophagy-animal, NOD-like receptor signaling pathway, osteoclast differentiation, regulation of actin cytoskeleton, Ribosome pathway (Figures 7A–J). The detailed enrichment results are deposited in https://www.ncbi.nlm.nih.gov/gds NCBI: GEO. Accession numbers are GSE16561 and GSE110993 respectively. Furthermore, the gene set variation of each sample in each specific pathway converted the new biological function annotation into a new expression matrix. GSVA analysis is shown in Figures 8A,B. There were differences in the grouping of terms such as KEGG-ribosome, GO BP-pyrimidine nucleotide biosynthetic process between the patients with acute cerebral infarction and the control group, or high and low expression groups, which were consistent with Figures 5A,B.

### Competitive Endogenous RNA Interaction Network Analysis, PPI Network Analysis

**FIGURE 9:**
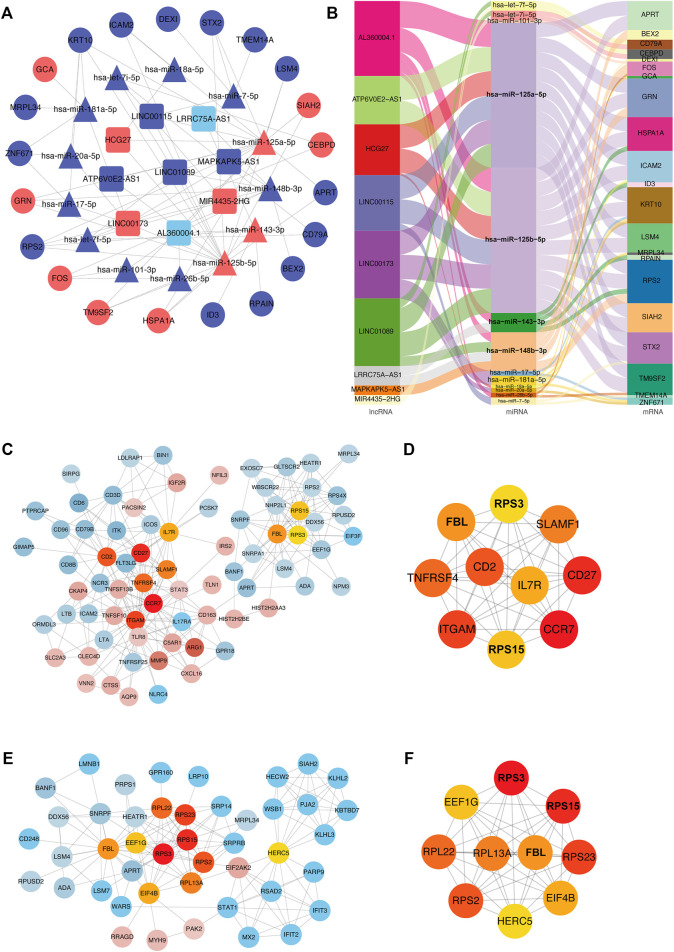
ceRNA interaction and protein-protein interaction analysis. **(A)** ceRNA network diagram. In the network diagram, red indicates upregulation, blue indicates downregulation, squares indicate lncRNA, triangles indicate miRNA, and circles indicate mRNA. Sankey diagram **(B)**. The three columns include lncRNAs, miRNAs, and mRNAs in order from left to right. The line colors represent different types of gene-gene interactions. **(C)** Diagram of interaction of differentially expressed proteins. Red indicates increased expression, blue indicates decreased expression, and color intensity indicates different degrees of u-regulation or downregulation. Orange represents the hub genes. **(D)** Diagram of hub-genes interaction in differentially expressed proteins. **(E)** MiRNAs targeting mRNAs interaction diagram. Red indicates upregulated expression, blue indicates downregulated expression, and color intensity indicates different degrees of upregulation or downregulation. Orange represents hub genes. **(F)** Diagram of hub genes interaction in targeted mRNAs.

We used the GDCRNATools package for data standardization, and then screened for differential mRNAs, miRNAs, and lncRNAs, including different forms of upregulation and downregulation, as shown in Figure 9A. Considering the number of miRNAs shared by mRNA, and lncRNA, key mRNAs were locked by combining with the miRcode database, as a database support for the interaction of lncRNA-miRNA and miRNA-mRNA. MiRNAs were identified through the mechanism of mRNA binding to ceRNA, and then a model was generated to evaluate the ceRNA interaction network, visualized using Cytoscape (Figure 9A). The ceRNA network was displayed also as a Sankey diagram (Figure 9B)**.** The network contained nine specific lncRNAs, 13 miRNAs, and 21 mRNAs. AL360004.1 (degree = 5),has-miR-125a-5p (degree = 16), hsa-miR-125b-5p (degree = 16), and KRT10 (degree = 5) were considered the most important transcripts among the lncRNAs, miRNAs, and mRNAs, respectively. Because hsa-miR-125a-5p, hsa-miR-125b-5p had the highest ceRNA degree (degree = 16), we concluded that this family gene might have an important influence on the pathogenesis of acute cerebral infarction. Next, we explored the interaction relationship between proteins encoded by different genes. PPI network of DEGs was established (Figure 9C), and the hub genes relationship was generated (Figure 9D). At the same time, according to the existing ceRNA network, interactions between differentially expressed miRNAs and their target genes were analyzed, as shown in Figure 9E, and its hub-genes are shown in Figure 9F.

### ROC Analysis of Key mRNA and miRNA

**FIGURE 10:**
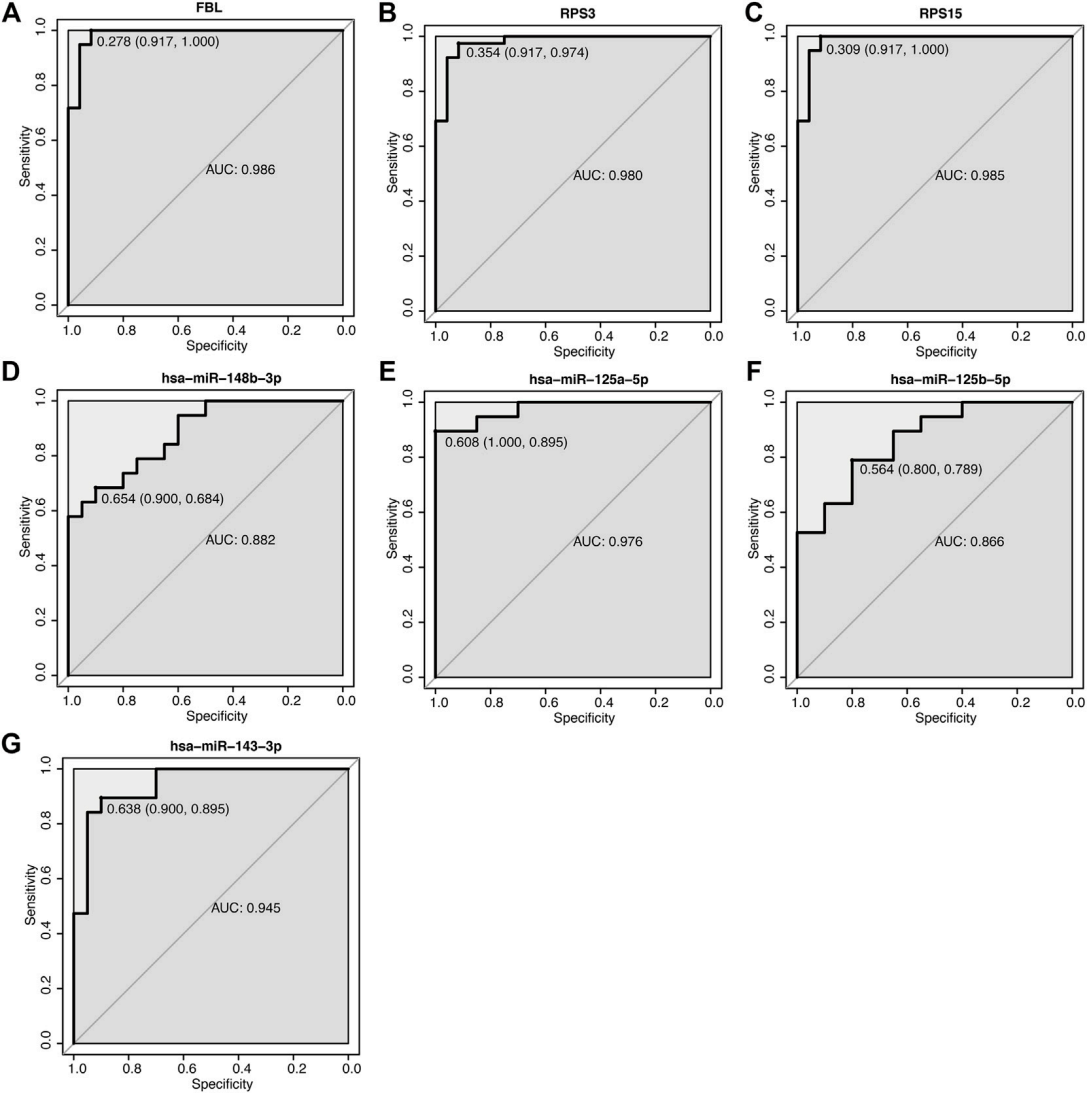
ROC curve of key mRNA and miRNA. **(A–G)** ROC curve of mRNA and miRNA. The abscissa is specificity, and the ordinate is sensitivity (true positive rate), specificity = 1 (false positive rate) AUC is the area under the ROC curve enclosed by the coordinate axis.

According to our previous ceRNA analysis (section 3.6), the molecules participating in the interaction network play a key role in the pathogenesis of acute cerebral infarction. Furthermore, we plotted the ROC curve by selecting the key mRNAs and miRNAs (Figures 10A–G), and then screened the biomarkers to reflect its predictive ability and accuracy for the disease.

### Immune Cell Infiltration Level and Correlation Analysis of Immune Cells

**FIGURE 11:**
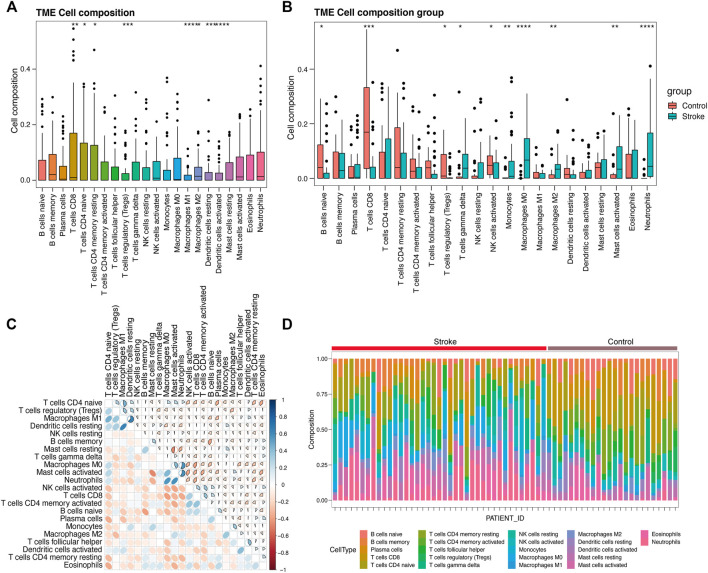
Evaluation and visualization of immune cell infiltration. **(A)** Ungrouped immune cell infiltration map. **(B)** Immune cell infiltration map between acute cerebral infarction group and control group. **(C)** Correlation heat map of 22 types of immune cell infiltration. Blue and red indicate positive and negative correlations, respectively. The darker the color, the stronger is the correlation. **(D)** Immune cell infiltration map between a single sample of acute cerebral infarction group and control group. *p < 0.05, **p < 0.01, ***p < 0.001, ****p < 0.0001.

By deconvolution analysis via CIBERSORT of the expression matrix of 21 immune cell subtypes were analyzed, using the limited threshold is *p* < 0.05. We obtained the immune cell infiltration matrix, and the results of immune infiltration distribution in acute cerebral infarction samples. Immune cell infiltration is shown in Figure 11A. Compared with other immune cells, T cells CD8, T cells CD4 naïve, and T cells CD4 memory resting were more infiltrated in samples that were not divided into acute cerebral infarction and control samples. However, Macrophage M and resting Dendritic cells infiltration is limited. After further grouping analysis, the significant infiltration of Macrophages M0, activated Mast cells, and Monocytes in the acute cerebral infarction samples difference compared with the control group (Figure 11B). In the control group, T cells CD8, B cells naïve, and activated NK cells had statistical increased in number compared with the acute cerebral infarction group. The next step was to analyze the correlation of different immune cells. As shown in Figure 11C, the proportions of different subgroups of infiltrating immune cells were weakly to moderately correlated. Neutrophils had a strong positive correlation with Macrophages M0 and activated Mast cells. T Cells CD8 were positively correlated with activated NK cells and B cells naïve, and Neutrophils were negatively correlated with T cells CD8. Figure 11D shows the changes in the proportion of immune cells within and between different groups. Therefore, the abnormal immune infiltration and the heterogeneity of immune infiltration in acute cerebral infarction suggested that different immune cells play a role in the occurrence and development of the disease. The immune signature may be used as prognostic targets for immunotherapy and may have significant clinical significance.

### Correlation Between Diagnostic Markers and Infiltrating Immune Cells

**FIGURE 12:**
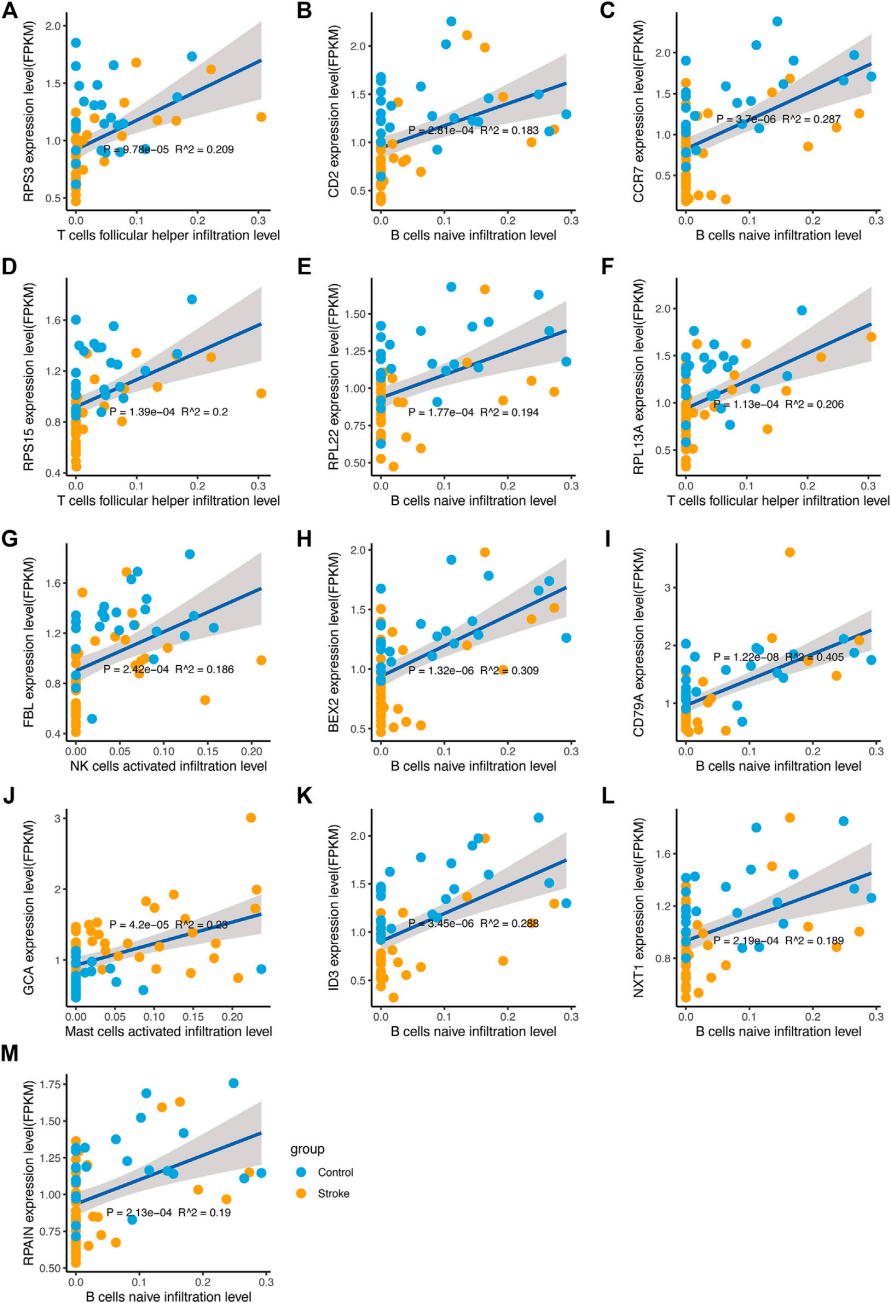
Correlation between diagnostic markers and immune cell infiltration. **(A–M)** The linear regression of diagnostic markers and immune cell infiltration level. The horizontal axis indicates the immune cell infiltration level, and the vertical axis indicates the marker expression. The *p* value is the regression significance level, and *R* ^2^ is the goodness-of-fit.

In the previous analysis, differentially expressed hub genes were selected, and ROC analysis was performed to screen for diagnostic markers. To determine the correlation between the hug genes and the immune cell infiltrations in acute cerebral infarction, linear regression was performed on the model, and the goodness-of-fit of regression model coefficients were evaluated to clarify the correlation between diagnostic markers and immune cell infiltration (Figure 12).

### Target Genes and Transcription Factor Network Analysis

**FIGURE 13:**
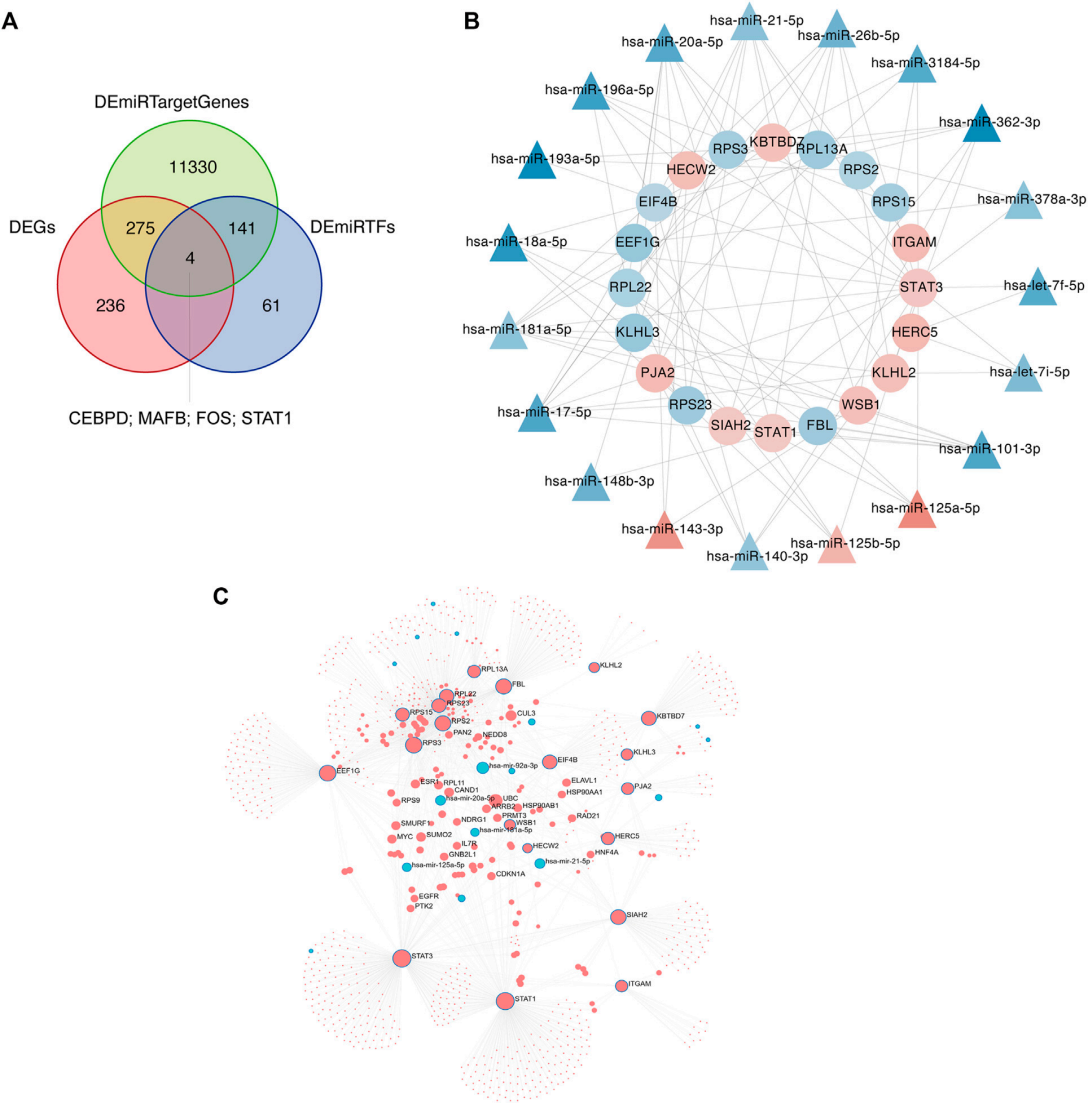
Network analyses of target genes and transcription factors. **(A)** The intersection of differentially expressed genes (DEGs), differential miRNA target genes, and transcription factors is determined using Venn diagram analysis. **(B)** Intersection molecules-miRNA network analysis. The inner ring is the intersection of DEGs and DEmiRTargetGenes, and the outer ring is miRNAs. Red indicates increased expression, blue indicates reduced expression, and color intensity indicates different degrees of upregulation or downregulation. **(C)** Network diagram of differential lncRNAs, miRNAs, and mRNAs. Red indicates upregulated expression; blue indicates downregulated expression.

Differential expression of mRNAs, miRNA target genes, and transcription factors were intersected to obtain four molecules, CEBPD, MAFB, FOS, and STAT1 (Figure 13A). Applying the intersection of DEGs and DEmiRTargetGenes as starting point, a network between these four molecules and miRNAs was generated (Figure 13B). The network integrating differential lncRNAs, miRNAs, and mRNAs shows the interaction analysis between target genes and transcription factors (Figure 13C **)**.

### Drug Sensitivity Analysis

**FIGURE 14:**
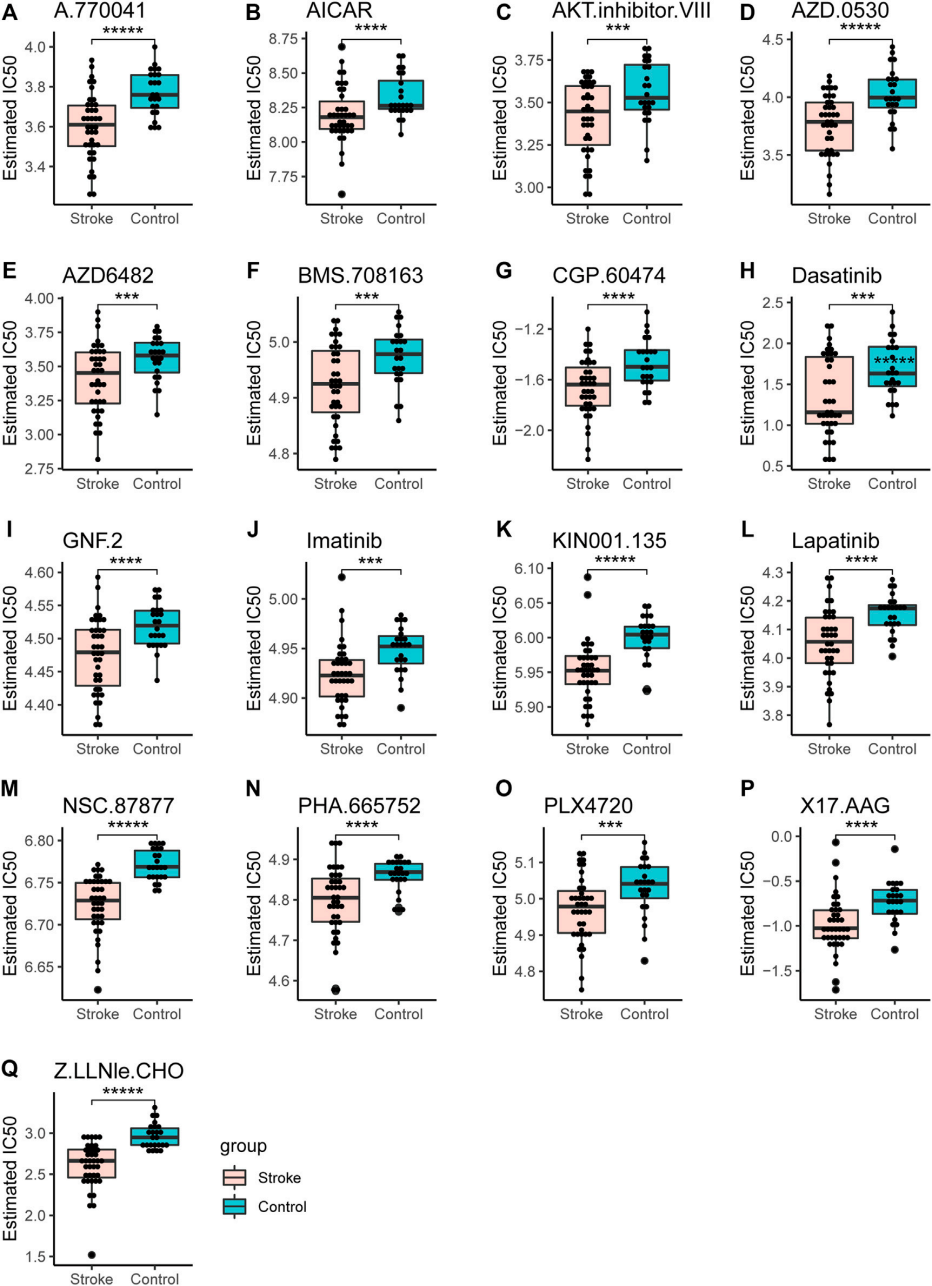
Drug sensitivity analysis. The IC50 values of different drugs were determined in the control group and the acute cerebral infarction group. *p < 0.05, **p < 0.01, ***p < 0.001, ****p < 0.0001, *****p < 0.00001.

The half-maximal inhibitory concentration (IC50), that is, the concentration of a drug that inhibits cell growth by 50% in different treatments, was predicted using the GDSC database. The value reflects the degree of cell tolerance to the drug. The lower the IC50 value, the more sensitive the cells are to drugs. IC50 and AUC values were obtained from all cell lines and drug combinations through the GDSC database. According to the screening results, we compared acute cerebral infarction with the control group and integrated the data. Finally, we visualized and predicted the IC50 of patients on drugs, and screened out drugs with significant differences between groups. (Figure 14).

### Validation of the Identified miRNAs and mRNAs

**FIGURE 15:**
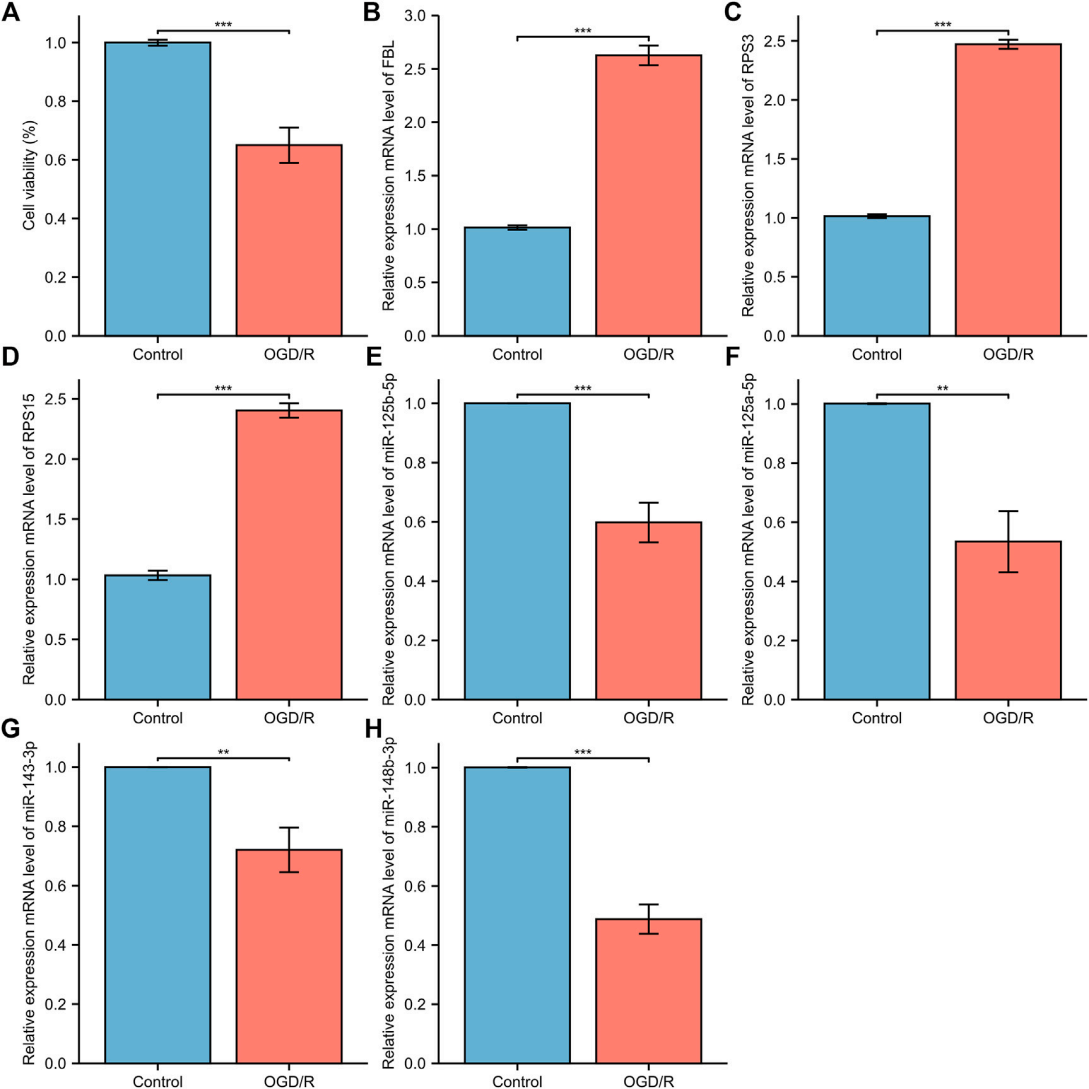
The relative expression of differentially expressed mRNA and miRNA in HT22. **(A)** The CCK-8 cell viability assay; **(B)** FBL **(C)** RPS3; **(D)** RPS15 **(E)** miRNA-143-3p **(F)** miRNA-148b-3p; **(G)** miRNA-125b-5p; **(H)** miRNA-125a-5p. The control group reflects the normal HT22 and the OGE/R group reflects the model group. *p < 0.05, **p < 0.01, ***p < 0.001.

Five Cell viability was assessed through the CCK-8 assay. Oxygen glucose deprivation/re-oxygenation (OGD/R) was used to mimic neural injury. Data demonstrated thatOGD/R insult in model group exhibited decreased cell viability, compared to the normal control group. When compared with the normal control group, the miRNA expression levels of miR-148b-3p (*p* < 0.01), miR-125a-5p (*p* < 0.05), miR-125b-5p (*p* < 0.01) and miR-143-3p (*p* < 0.05) in the model control group were significantly downregulated while the mRNA expression levels of FBL (*p* < 0.01), RPS3 (*p* < 0.01), and RPS15 (*p* < 0.01) were significantly upregulated (Figure 15).

## Discussion

The incidence of AIS increases with aging and is linked to a poor prognosis. Timely monitoring of undiagnosed strokes is critical to reduce mortality. However, long delays in imaging and treatment initiation and poor functional prognosis represent a significant challenge. Despite remarkable advances in the development of therapeutic strategies, developing effective targeted molecular therapies is limited. Therefore, there has been an increased search for noninvasive and quantitative markers for AIS. This study aimed to identify novel biomarkers with sensitivity and specificity for the diagnosis and treatment of AIS. Through a comprehensive analysis, a total of 3698 DEGs were identified from the mRNA microarray dataset (GSE16561) and a total of 26 DE-miRNAs were identified from the miRNA high-throughput sequencing dataset (GSE110993). GO enrichment, KEGG pathway analysis, GSEA and GSVA were performed to enrich and analyze DEGs. We performed ceRNA interactions analysis and a network of miRNA-mRNA interactions in patients with acute cerebral infarction, allowing us to uncover potential biomarkers associated with AIS. A total of 17 hub genes were identified based on DEGs and miRTargetgenes. We compared the levels of immune cell infiltrates in cerebral infarction group and control group, and we found that screened diagnostic markers correlated with immune cell infiltration. We also identified drugs with significant differences between groups by predicting the patient’s IC50 for the drug.

First, functional annotation to DEGs displayed enrichment of GO and KEGG pathway analyses referring to inflammation and immune response. Biological processes analysis in GO annotation indicated that DEGs were primarily enriched in the inflammatory response associated with neutrophils, T cell and lymphocytes. Among them, neutrophils are involved in multiple biological processes, including neutrophil activation, neutrophil degranulation, and neutrophil activation. Neutrophils have received particular attention during recent years of their significant destructive potential. Experimental studies have shown that neutrophils reach the ischemic area in the first few hours after an ischemic attack (Perez-de-Puig et al., 2015). They can release neurotoxic proteolytic enzymes, accumulate neutrophils in blood vessels, block blood flow in capillaries and cause the no-reflow phenomenon (Allen et al., 2012; Perez-de-Puig et al., 2015).In terms of molecular function annotations, the occurrence of AIS was closely related to amide binding, peptide binding, structural constituent of ribosome, and amyloid-beta binding. KEGG suggested that most of DEGs in subjects were mainly enriched in the toll-like receptor signaling pathway, Cell adhesion molecules, T cell receptor signaling pathway, NF−kappa B signaling pathway, the B cell receptor signaling pathway, which have collectively been confirmed as essential mechanisms in inflammation of ischemic stroke. The theranostic strategy is a combination of diagnosis and therapy which can be furnished through analyzing relevant data in the GEO database and forming an AIS-related lncRNA-miRNA-mRNA regulatory network.

To detect underlying biological functions, GSEA and GSVA were performed. The results of GSEA suggest that the NOD-like receptor signaling pathway and autophagy pathway were significantly enriched pathways. In cerebral ischemic injury, autophagy can be protective (Carloni et al., 2010) or destructive (Koike et al., 2008). If its protective function can be controlled, autophagy may become novel therapeutic targets for ischemic brain (Carloni et al., 2010). Moreover, NOD-like receptor, which regulates innate immunity and inflammatory processes (Shiau et al., 2013), is expected to become as a therapeutic target in ischemic stroke. GSVA analysis confirmed that the most abundant pathways are related to immune response, inflammation and apoptosis.

Using GEO, differentially expressed transcripts, including mRNAs, miRNAs and lncRNAs, were identified and the ceRNA network in AIS was constructed. In the ceRNA network, AL360004.1 (degree = 5), hsa-miR-125a-5p (degree = 16), hsa-miR-125b-5p (degree = 16) and KRT10 (degree = 5) were the molecules with the highest connection score. Previous studies have correlated cancer-related functions with AL360004.1, LINC00173, LINC01089 and LINC00115; however, none of them have been linked to the pathogenesis of AIS. LINC00115 expression levels correlate with prognosis in human bladder (Jiang et al., 2018) and lung cancer (Li et al., 2016) patients. A study demonstrated that LINC00115, a novel miRNA sponge of the miR-200 family, can promote ZEB1 signaling in GBM (Tang et al., 2019). Deletion or ectopic expression of LINC00115 affects ZEB1 signaling, neuro-like sphere formation *in vitro*, and animal survival time *in vivo* (Tang et al., 2019). A study reported that LINC00173 plays an important oncogenic role in glioma by activating the miR-765/NUTF2 pathway (Du et al., 2020). And silencing of LINC00173. V1 attenuates vascular endothelial cell proliferation and migration (Chen J. et al., 2020). Thus, they represent a potential novel biological markers for AIS diagnosis and therapy. KRT10 is an intermediate filament (IF) protein belonging to the type I (acidic) cytokeratin family. Keratin expression may affect cell proliferation and differentiation (Paladini and Coulombe, 1998; Paramio et al., 1999). KRT10 impairs cell cycle progression through isolating and inhibiting protein kinase B (PKB; Akt) and atypical PKC, the key effectors of the phosphatidylinositol 3-kinase (PI3K) pathway (Paramio et al., 2001). PI3K pathway plays a central role in neuronal survival. Therefore, KRT10 may also be a potential therapeutic targets for AIS. MiR-125a and miR-125b belong to the same miRNA family, which has identical ‘seed sequence’. MiR-125a-5p and miR-125b-5p both have been shown to inhibit angiogenesis (Banerjee et al., 2013; Pan et al., 2015). Overexpression of miR-125a-5p promotes nitric oxide (NO) production, reduces ROS production, and delays human brain microvascular endothelial cells (HBMECs) senescence through the PI3K/AKT/eNOS signaling pathway (Pan et al., 2017). MiR-125b-5p regulates synaptic morphology and function (Edbauer et al., 2010). MiR-125a-5p is associated with colorectal and liver cancer diseases (Chen L.-Y. et al., 2020; Zhou et al., 2021). Acute kidney injury and triple-negative breast cancer are associated with miR-125b-5p (Lv et al., 2021). According to a report, MiR-148b plays multiple roles in the development of various biological processes (Friedrich et al., 2017). A study confirmed the role of miR-148b in the modulation of proliferation and differentiation of neural stem cell after ischemic stroke (Wang et al., 2017). Therefore, we determined that this group of miRNAs is a promising diagnostic marker. Seventeen DEGs were highly connected as the most significant hub genes in the PPI network and there are multiple interactions in the network. Moreover, FBL, RPS3 and RPS15 were ranked as the top three proteins of the most potentially serving as key regulators in AIS. Both RPS3 and RPS15 encode a ribosomal protein, which is part of the 40S subunit. Interestingly, GO and KEGG analysis, as well as GSEA and GSVA, showed that ribosome pathway was closely related to the increased incidence of ischemic stroke. RPS3 induces neuronal apoptosis by interacting with the E2F1 transcription factor and inducing the expression of pro-apoptotic proteins BCL2L11/BIM and HRK/Dp5 (Lee et al., 2010). When located in the mitochondria, RPS3 reduces cellular ROS levels and mitochondrial DNA damage (Kim et al., 2013). Diseases associated with RPS3 include Eumycotic Mycetoma and Schopf-Schulz-Passarge Syndrome. A study shows that RPS15 is a critical morbific leucine-rich repeat kinase 2 (LRRK2) substrate in Parkinson’s disease models of *drosophila* and human neuron (Martin et al., 2014). Phosphorylation of RPS15 is related to LRRK2 neurodegeneration and neurotoxicity (Martin et al., 2014), suggesting that the genes encoding ribosomal proteins may be potential targets and treatment for early diagnosis of AIS. The components of small nucleolar ribonucleoprotein (snRNP) particles include FBL products, which are required for ribosomal RNAs processing and modification. FBL interacts with small nucleolar RNAs (snoRNAs) and ribosomal RNA (rRNA) to modify the mRNA 2′-O-methylation, thereby regulating ROS and oxidative reactions (Elliott et al., 2019). Diseases associated with FBL include Diffuse Scleroderma and Systemic Scleroderma. In terms of diagnostic value, the AUC of these three mRNAs and four miRNAs’ genes was analyzed. All the AUC values were in the range 0.866–0.989, suggesting that these genes had moderate predictor performances (Akobeng, 2007) in diagnostic examinations. However, in our investigation the qPCR results showed that miR-125a-5p, miR-125b-5p, and miR-143-3p levels in the model control group were significantly downregulated. Thus, these results drive us to explore whether these miRNAs will also play a protective role in AIS when cells viability is around 65%. The validation of identified DEGs and DEmiRs was performed using qPCR, and confirmed the above results, proof of the diagnostic effectiveness of the DEGs and DEMirs.

It was further confirmed that the infiltration levels of Macrophages M0, activated Mast cells, and Monocytes in acute cerebral infarction samples were higher than those in control samples. Contrarily, the infiltration levels of T cells CD8, B cells naïve, and activated NK cells in control samples significantly higher than those in AIS samples. Macrophages infiltrate and promote rapid inflammatory responses in the acute phase of AIS; however, T cells in the late phases of cerebral infarction (Iadecola and Anrather, 2011). Animal models of ischemic stroke results in increased number of activated mast cells (Bot et al., 2020), and mast cells are also involved in arteriogenesis and collateral formation (Chillo et al., 2016). Activation of mast cells plays a proinflammatory role by recruiting immune cells such as neutrophils and monocytes (Bot et al., 2007; Sun et al., 2007; Chillo et al., 2016). After identifying differential expression of hub-genes, we analyzed the correlation between the hub genes of AIS and the level of immune infiltration. It has been suggested that peripheral immune cells such as neutrophils to infiltrate in the ischemic brain region after disruption of the blood-brain barrier (BBB) in ischemic stroke (Qian et al., 2016). In addition, elevated expression level of hub-genes significantly correlated with T cells follicular helper, B cells naive, NK cells, and Mast cells infiltration (*p* < 0.05), facilitating a general increase the levels of infiltrating immune cells.

Four transcription factors were obtained by taking the intersection of DEmRNA, DemiRTargetGenes, and DEmiRTFs:CEBPD, MAFB, FOS, and STAT1. CEBPD is an important TF that regulates the expression of multiple genes and participates in immune and inflammatory responses (Wang et al., 2006). MAFB avoids excess inflammation after ischemic stroke (Shichita et al., 2017). FOS plays a s crucial role in post ischemic inflammation and cell death (Chung, 2015). STAT1 is activated by ROS and contributes to ischemic injury (Takagi et al., 2002). AZD0530 is a small molecule inhibitor of Src family kinases under investigation. Moreover, orally administered AZD0530 is highly CNS penetrable in both mice and humans (Kaufman et al., 2015). Recent a study shows that AZD0530 rescues deficits in memory and restores synapse density in transgenic mouse Alzheimer disease models (van Dyck et al., 2019). Elevated or stable Notch levels can promote neuronal death in ischemic stroke. However, NOTCH signaling pathway is inhibited through a gamma-secretase inhibitor (GSI) (Xu et al., 2016). On the other hand, GSIs have been used for the treatment of Alzheimer’s disease to prevent the cleavage of amyloid precursor protein and the subsequent release of amyloid *β* peptide. Therefore, this suggests that AZD0530 and GSI-I (Z.LLNle.CHO) can be used as therapeutic agents in models of Alzheimer’s disease. Whether they can be used as a therapeutic agent for cerebral infarction remains unclear. NSC-87877 is an effective Shp2 inhibitor, but it has a similar inhibitory effect on Shp1. Some studies have reported that NSC-87877 is a potential new treatment for relapsing-remitting multiple sclerosis, MuSK antibody positive myasthenia gravis (MuSK-MG) (Huda et al., 2020) and intracerebral hemorrhage (Liu et al., 2019). In addition, Yinlong et al. reported that NSC 87877 treatment attenuated ICH-induced apoptosis and neuronal death (Liu et al., 2019). In this regard, we speculated whether NSC-87877 could not only promote cerebral neovascularization and the brain vascular restoration after stroke but also exert neuroprotective effects.

Our study presents few limitations. First, a comprehensive analysis of warranted venous blood samples and brain tissue was not performed in this study; however, it is necessary to comprehensively diagnose the dysfunctions in acute ischemic stroke. Second, the study includes a relatively small cohort and, therefore, some of the data failed to reach statistical significance. To determine better accuracy and validation of the hub genes associated with AIS, a larger sample size for further external validation is needed. Third, the results should be further verified by western blot (WB), real-time PCR and immunofluorescence assays. Further experiments are clearly warranted to fully elucidate the role of hub genes and the underlying mechanisms of acute ischemic stroke. Fourth, to investigate potential function and mechanisms related to DEGs and hub genes in AIS, the study of the cell or tissue-type specific gain-of-function and loss-of-function still needs to be performed. Signaling pathways are more diverse in AIS than previously thought, such as Toll-like receptor 4 (TLR4)/NF-κB/NLRP3 signaling pathway, T cell receptor signaling pathway, and PPAR-γ signaling pathway. Although previous studies have identified several signaling pathways, more detail experimental evidence is still needed to improve our understanding of the possible phenotype and pathway regulation of these predicted genes in AIS. Fifth, ceRNA network and interaction among hub genes will be an exciting new field to explore and will shed new light on ischemic cerebrovascular disease. Co-Immunoprecipitation and pull-down assays would provide strong support to the proposed mechanism. Further investigation is needed to explore the intermolecular interactions responsible for the molecular cooperativity in the progression of cerebral infarctions, such as the ribosomal protein family (C and D). Moreover, the contribution of the identified DEGs into the pathogenesis of AIS should be examined in detail. Therefore, it may be necessary to test the efficacy of activation and inactivation in more experiments to molecular interactions. These are very valuable in understanding the mechanisms of protein-protein interactions. Sixth, ArenaIdb database (Bonnici et al., 2018) truly integrates the content of starbase, mircode with other datasets that the authors have not used. We do not currently do analysis of other datasets using arenaidb. In the follow-up research, we will supplement the validation of other datasets.

In conclusions, this study identified several pathways and biomarkers in AIS consistent with current knowledge of the pathology of this disease. We believe that new insights are provided on the molecular mechanisms underlying the pathogenesis of AIS.

## Data Availability

The datasets presented in this study can be found in online repositories. The names of the repository/repositories and accession number(s) can be found in the article/Supplementary Material.
