# Supplementary material for: Integrated Analysis of Competitive Endogenous RNA Networks in Acute Ischemic Stroke
Source: Front Genet. 2022 Mar 25;13:833545. doi: 10.3389/fgene.2022.833545 (PMC8990852; doi:10.3389/fgene.2022.833545)
Supplement: Supplementary file 3 [file Table1.DOCX]

**Supplementary Table 1 PCR primers used in this study**

| **Primer name** | **Sequence** |
| --- | --- |
| FBL | F: 5’-CAGGAGCCAAGGTGCTCTAC-3’ |
|  | R: 5’-GGGCCGACAATATCAGAGAC-3’ |
| RPS3 | F: 5’-CTGGGACCCAAGTGGTAAGA-3’ |
|  | R: 5’-TTCCACGATGCTCACATGAT-3’ |
| RPS15 | F: 5’-GAAGCCTGAGGTGGTGAAGA-3’ |
|  | R: 5’-GTTGAAGGTCTTGCCGTTGT-3’ |
| hsa-miR-148b-3p | F: 5’-TCAGTGCATCACAGAACTTTGT-3’ |
| hsa-miR-125a-5p | F: 5’-TCCCTGAGACCCTTTAACCTGTGA-3’ |
| hsa-miR-125b-5p | F: 5’-TCCCTGAGACCCTAACTTGTGA-3’ |
| hsa-miR-143-3p | F: 5’-TTGGGAGTCTGAGATGAAGC-3’ |

F：Forward primer；R：Reverse primer。
