# Supplementary material for: Integrated Analysis of Competitive Endogenous RNA Networks in Acute Ischemic Stroke
Source: Front Genet. 2022 Mar 25;13:833545. doi: 10.3389/fgene.2022.833545 (PMC8990852; doi:10.3389/fgene.2022.833545)
Supplement: Supplementary file 5 [file DataSheet2.ZIP › Supplementary Table 3/GO_ACUTE_PHASE_RESPONSEGSVA_ Supplementary Table 3.pdf]

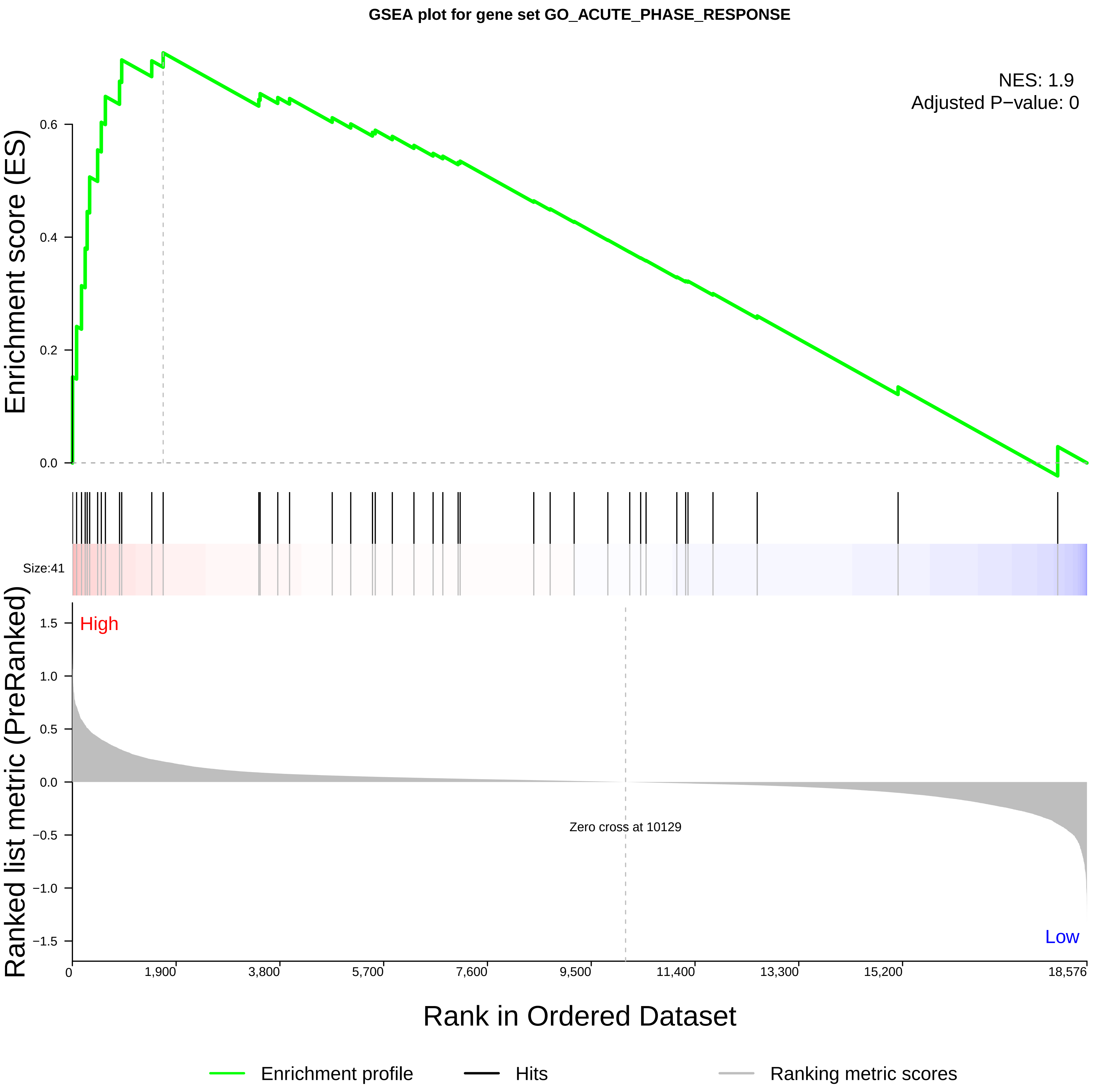
GSEA plot for gene set GO_ACUTE_PHASE_RESPONSE
NES: 1.9
Adjusted P−value: 0
Enrichment score (ES)
0.6
0.4
0.2
0.0
Size:41
Ranked list metric (PreRanked)
High
Low
1.5
1.0
0.5
0.0
−0.5
−1.0
−1.5
Zero cross at 10129
0
1,900
3,800
5,700
7,600
9,500
11,400
13,300
15,200
18,576
Rank in Ordered Dataset
Enrichment profile
Hits
Ranking metric scores
