# Supplementary figures and images for: Integrated Analysis of Competitive Endogenous RNA Networks in Acute Ischemic Stroke
Source: Front Genet. 2022 Mar 25;13:833545. doi: 10.3389/fgene.2022.833545 (PMC8990852; doi:10.3389/fgene.2022.833545)

# GSEA plot for gene set GO\_ACTIN\_FILAMENT\_BASED\_PROCESS

NES: 1.9  
Adjusted P-value: 0

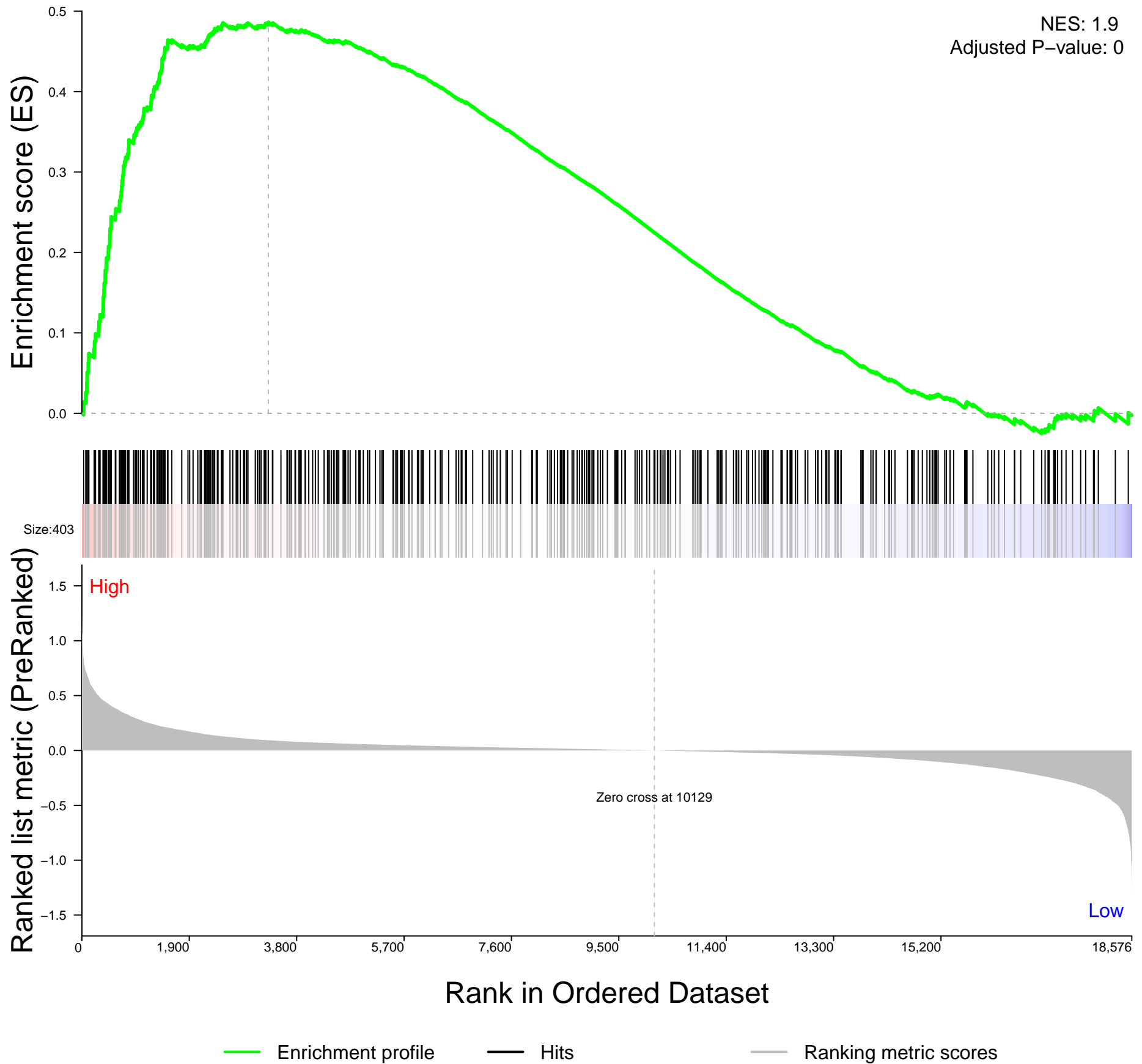

Supplement: Supplementary file 5 [file DataSheet2.ZIP › Supplementary Table 3/GO_ACTIN_FILAMENT_BASED_PROCESSGSVA_ Supplementary Table 3.pdf]

GSEA plot for gene set GO\_ACTIN\_FILAMENT\_BUNDLE\_ORGANIZATION

NES: 1.9  
Adjusted P-value: 0

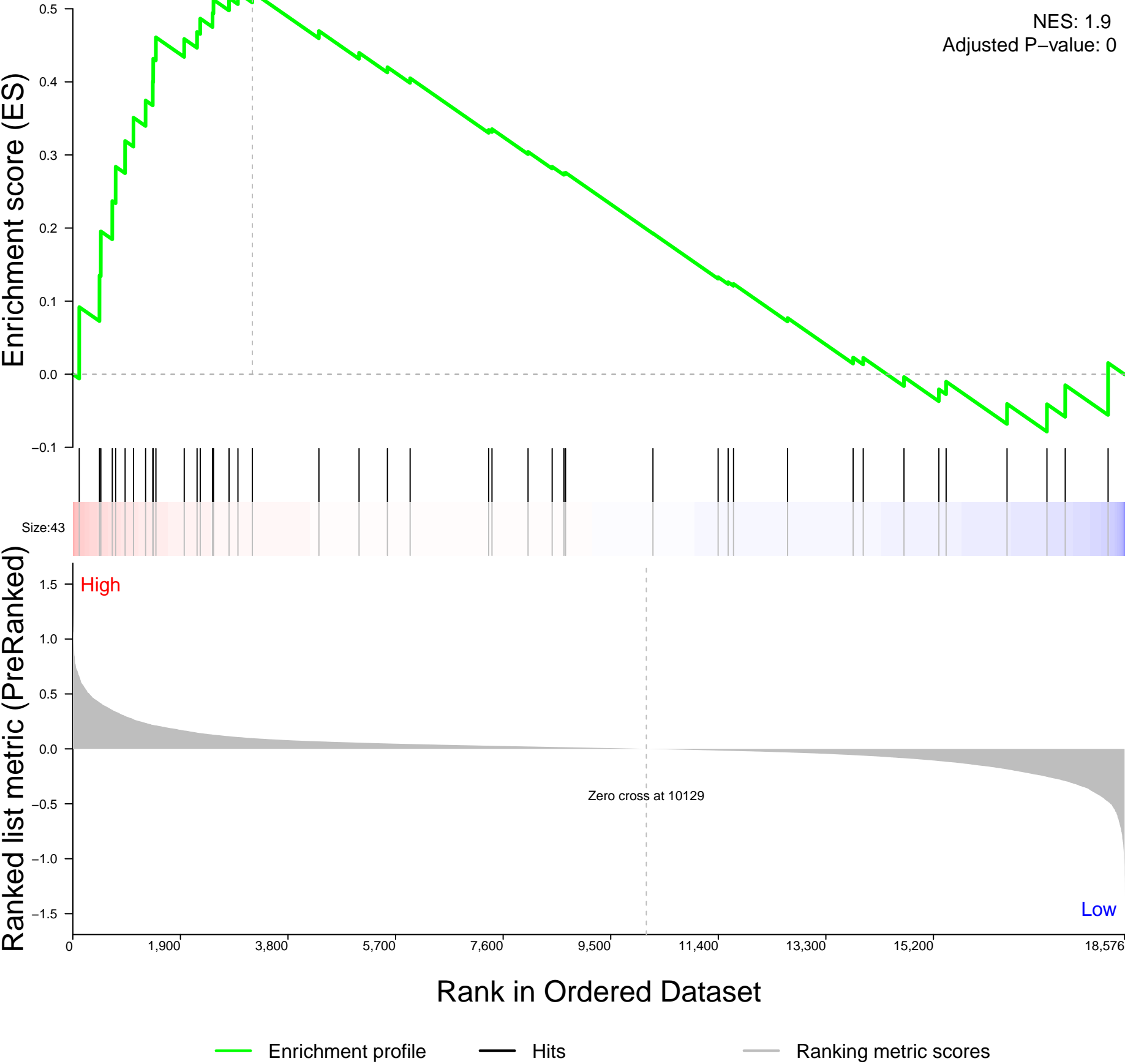

Supplement: Supplementary file 5 [file DataSheet2.ZIP › Supplementary Table 3/GO_ACTIN_FILAMENT_BUNDLE_ORGANIZATIONGSVA_ Supplementary Table 3.pdf]

# GSEA plot for gene set GO\_ACTIN\_FILAMENT\_ORGANIZATION

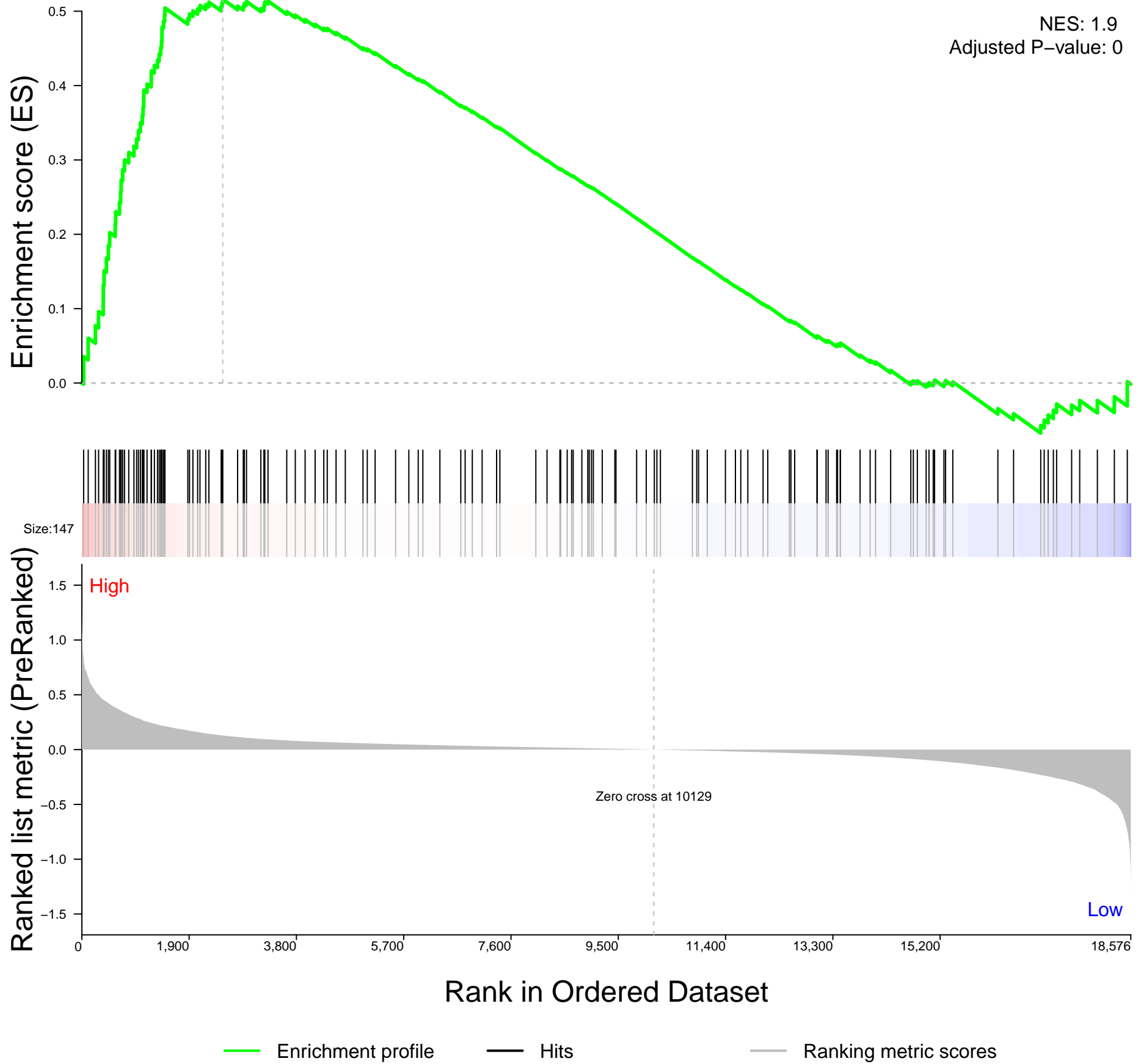

Supplement: Supplementary file 5 [file DataSheet2.ZIP › Supplementary Table 3/GO_ACTIN_FILAMENT_ORGANIZATIONGSVA_ Supplementary Table 3.pdf]

# GSEA plot for gene set GO\_ACTIN\_FILAMENT\_POLYMERIZATION

NES: 1.9  
Adjusted P-value: 0

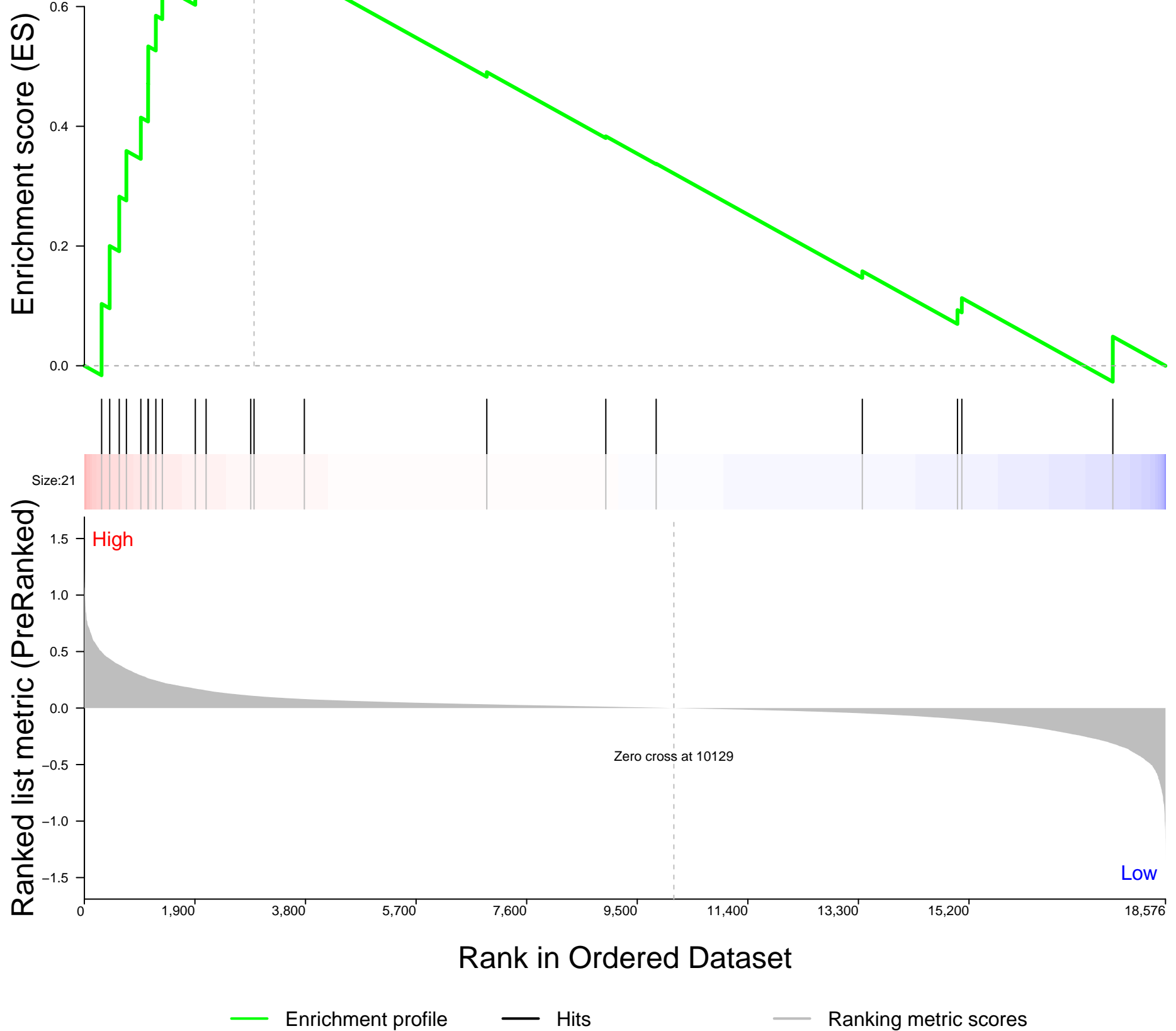

Supplement: Supplementary file 5 [file DataSheet2.ZIP › Supplementary Table 3/GO_ACTIN_FILAMENT_POLYMERIZATIONGSVA_ Supplementary Table 3.pdf]

# GSEA plot for gene set GO\_ACTIN\_MEDIATED\_CELL\_CONTRACTION

NES: 1.9  
Adjusted P-value: 0

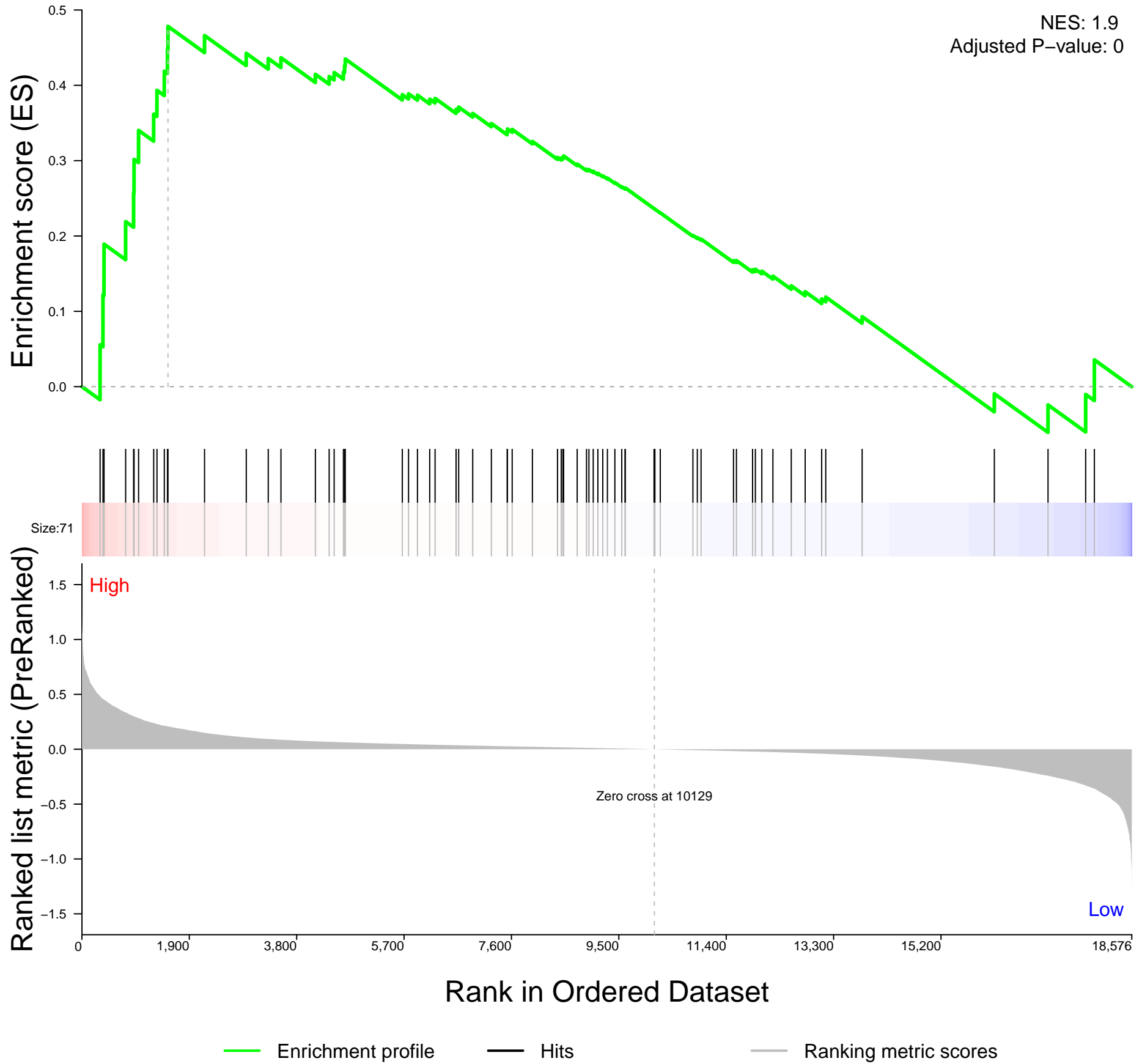

Supplement: Supplementary file 5 [file DataSheet2.ZIP › Supplementary Table 3/GO_ACTIN_MEDIATED_CELL_CONTRACTIONGSVA_ Supplementary Table 3.pdf]

# GSEA plot for gene set GO\_ACTIN\_POLYMERIZATION\_OR\_DEPOLYMERIZATION

NES: 1.9  
Adjusted P-value: 0

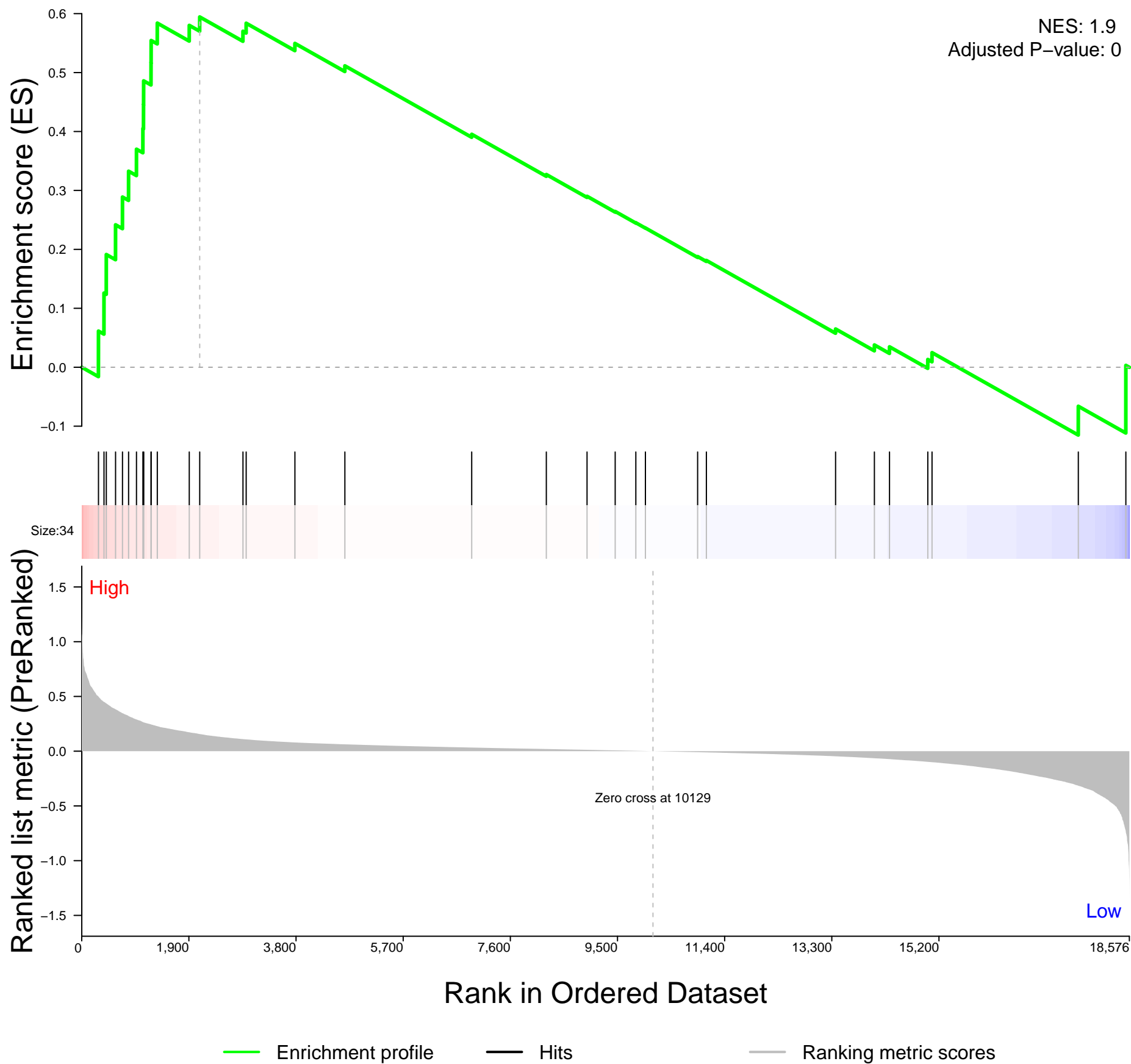

Supplement: Supplementary file 5 [file DataSheet2.ZIP › Supplementary Table 3/GO_ACTIN_POLYMERIZATION_OR_DEPOLYMERIZATIONGSVA_ Supplementary Table 3.pdf]

GSEA plot for gene set GO\_ACTIVATION\_OF\_IMMUNE\_RESPONSE

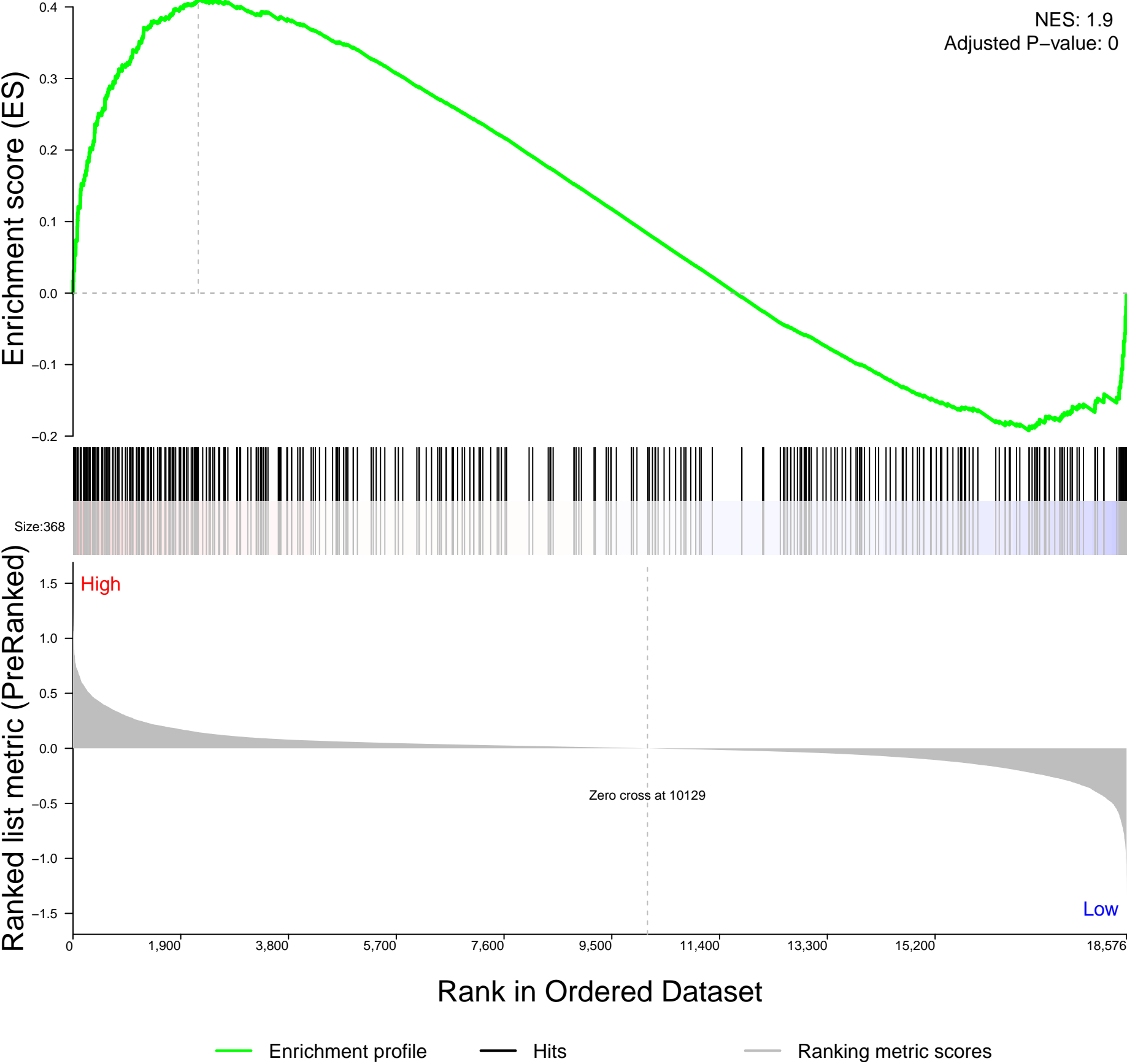

Supplement: Supplementary file 5 [file DataSheet2.ZIP › Supplementary Table 3/GO_ACTIVATION_OF_IMMUNE_RESPONSEGSVA_ Supplementary Table 3.pdf]

# GSEA plot for gene set GO\_ACTIVATION\_OF\_INNATE\_IMMUNE\_RESPONSE

NES: 1.9  
Adjusted P-value: 0

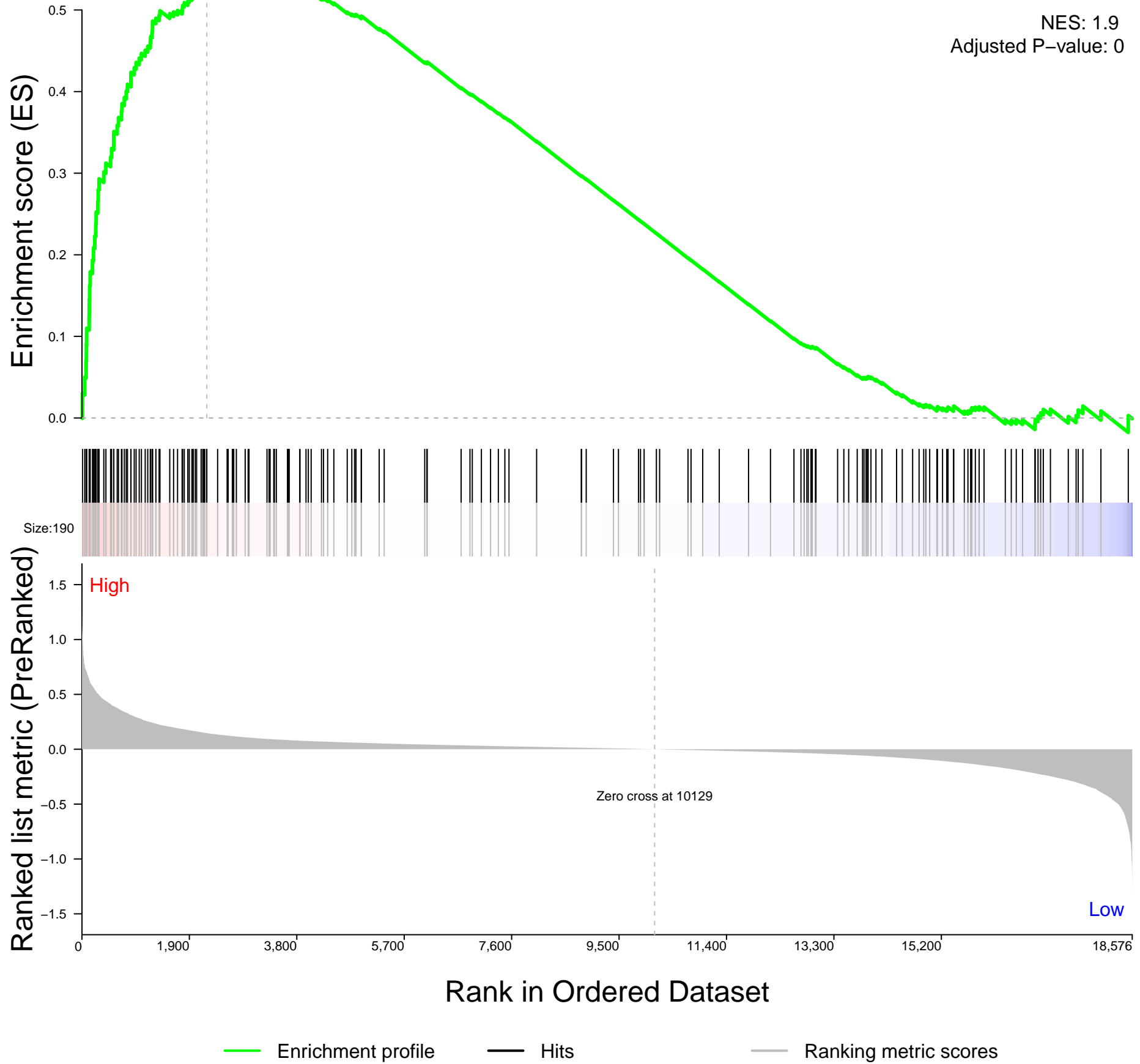

Supplement: Supplementary file 5 [file DataSheet2.ZIP › Supplementary Table 3/GO_ACTIVATION_OF_INNATE_IMMUNE_RESPONSEGSVA_ Supplementary Table 3.pdf]

# GSEA plot for gene set GO\_ACTIVATION\_OF\_MAPKK\_ACTIVITY

NES: 1.9  
Adjusted P-value: 0

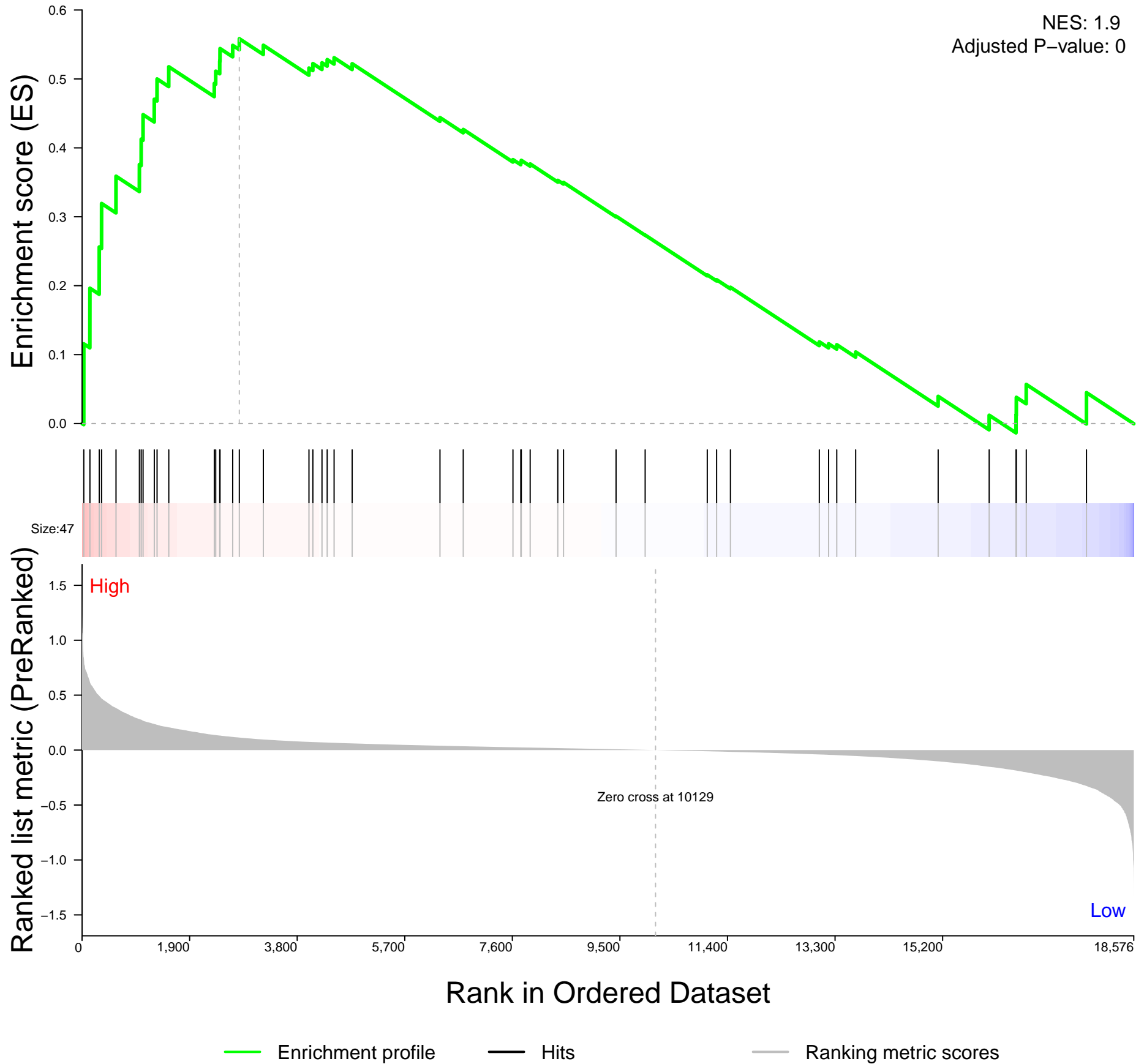

Supplement: Supplementary file 5 [file DataSheet2.ZIP › Supplementary Table 3/GO_ACTIVATION_OF_MAPKK_ACTIVITYGSVA_ Supplementary Table 3.pdf]

# GSEA plot for gene set GO\_ACTIVATION\_OF\_PROTEIN\_KINASE\_ACTIVITY

NES: 1.9  
Adjusted P-value: 0

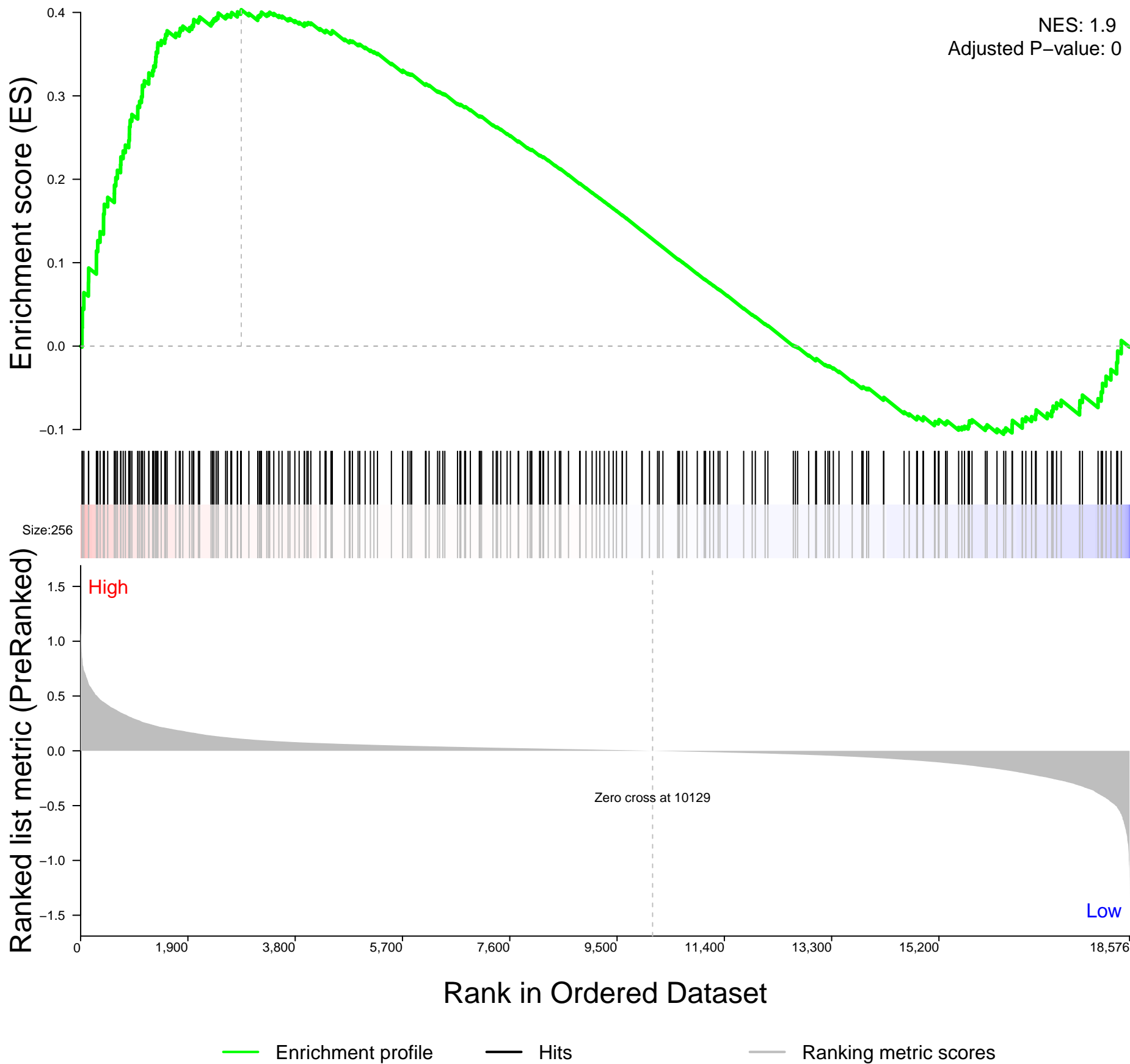

Supplement: Supplementary file 5 [file DataSheet2.ZIP › Supplementary Table 3/GO_ACTIVATION_OF_PROTEIN_KINASE_ACTIVITYGSVA_ Supplementary Table 3.pdf]

# GSEA plot for gene set GO\_ACUTE\_INFLAMMATORY\_RESPONSE

NES: 1.9  
Adjusted P-value: 0

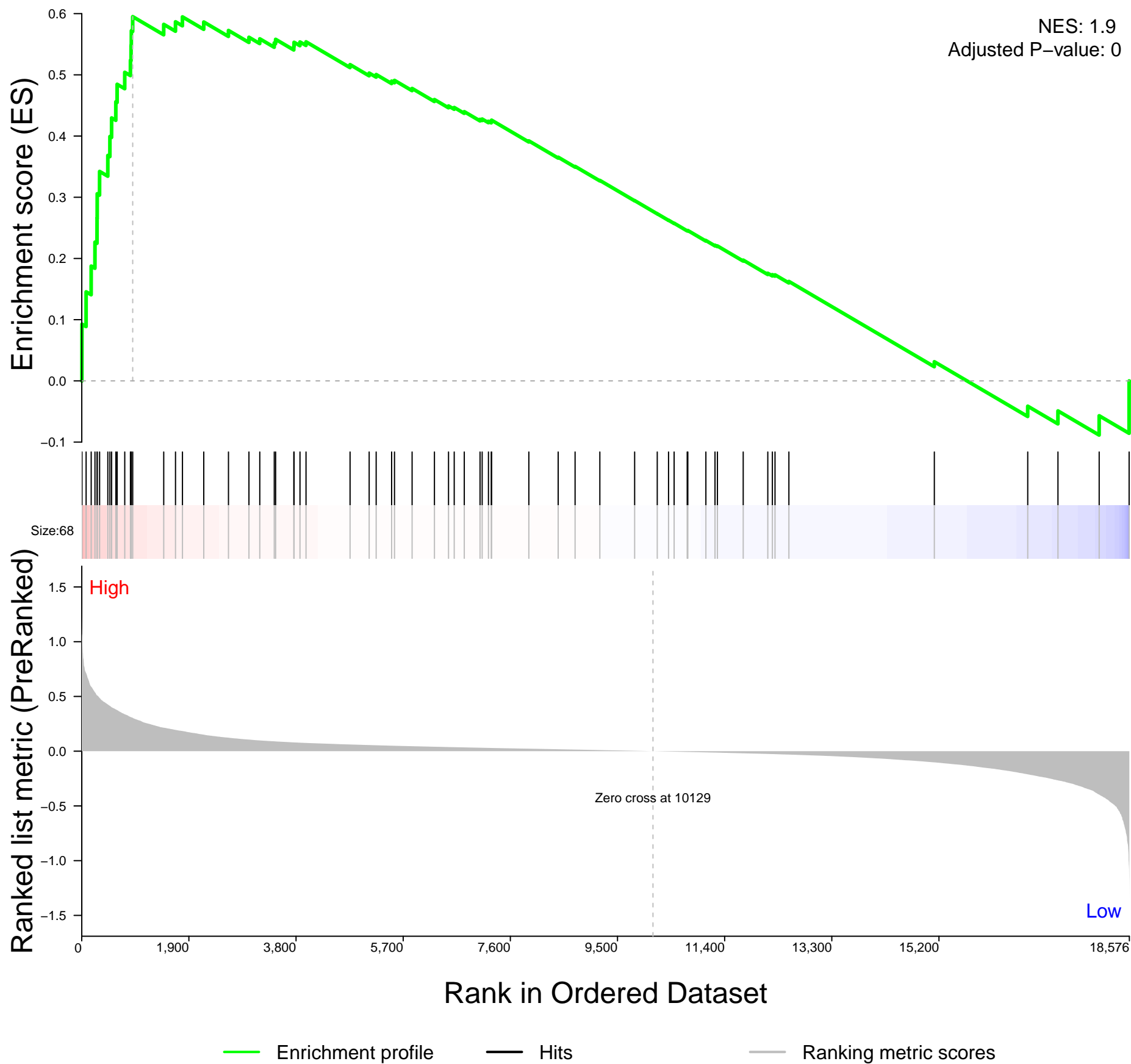

Supplement: Supplementary file 5 [file DataSheet2.ZIP › Supplementary Table 3/GO_ACUTE_INFLAMMATORY_RESPONSEGSVA_ Supplementary Table 3.pdf]
